# Transcriptomic study of pedicels from GA_3_-treated table grape genotypes with different susceptibility to berry drop reveals responses elicited in cell wall yield, primary growth and phenylpropanoids synthesis

**DOI:** 10.1186/s12870-020-2260-6

**Published:** 2020-02-10

**Authors:** Marco Meneses, Miguel García-Rojas, Claudia Muñoz-Espinoza, Tomás Carrasco-Valenzuela, Bruno Defilippi, Mauricio González-Agüero, Claudio Meneses, Rodrigo Infante, Patricio Hinrichsen

**Affiliations:** 1 Instituto de Investigaciones Agropecuarias, INIA-La Platina, Santa Rosa 11610, Santiago, Chile; 2 Programa de Doctorado en Ciencias Silvoagropecuarias y Veterinarias, Campus Sur, Universidad de Chile, Santiago, Chile; 3 Centro de Biotecnología Vegetal, Facultad de Ciencias de la Vida, Universidad Andrés Bello, Santiago, Chile; 4 FONDAP Center for Genome Regulation, Universidad Andres Bello, Santiago, Chile

**Keywords:** Gibberellic acid, Berry drop, RNAseq, *Vitis vinifera* L., Shattering, Postharvest, pedicel

## Abstract

**Background:**

Gibberellins (GA_3_) are the most sprayed growth regulator for table grape production worldwide, increasing berry size of seedless varieties through pericarp cell expansion. However, these treatments also exacerbate berry drop, which has a detrimental effect on the postharvest quality of commercialized clusters. Several studies have suggested that pedicel stiffening caused by GA_3_ would have a role in this disorder. Nevertheless, transcriptional and phenotypic information regarding pedicel responses to GA_3_ is minimal.

**Results:**

Characterization of responses to GA_3_ treatments using the lines L23 and Thompson Seedless showed that the former was up to six times more susceptible to berry drop than the latter. GA_3_ also increased the diameter and dry matter percentage of the pedicel on both genotypes. Induction of lignin biosynthesis-related genes by GA_3_ has been reported, so the quantity of this polymer was measured. The acetyl bromide method detected a decreased concentration of lignin 7 days after GA_3_ treatment, due to a higher cell wall yield of the isolated fractions of GA_3_-treated pedicel samples which caused a dilution effect. Thus, an initial enrichment of primary cell wall components in response to GA_3_ was suggested, particularly in the L23 background. A transcriptomic profiling was performed to identify which genes were associated with these phenotypic changes. This analysis identified 1281 and 1787 genes differentially upregulated by GA_3_ in L23 and cv. Thompson Seedless, respectively. Concomitantly, 1202 and 1317 downregulated genes were detected in L23 and cv. Thompson Seedless (FDR < 0.05). Gene ontology analysis of upregulated genes showed enrichment in pathways including phenylpropanoids, cell wall metabolism, xylem development, photosynthesis and the cell cycle at 7 days post GA_3_ application. Twelve genes were characterized by qPCR and striking differences were observed between genotypes, mainly in genes related to cell wall synthesis.

**Conclusions:**

High levels of berry drop are related to an early strong response of primary cell wall synthesis in the pedicel promoted by GA_3_ treatment. Genetic backgrounds can produce similar phenotypic responses to GA_3_, although there is considerable variation in the regulation of genes in terms of which are expressed, and the extent of transcript levels achieved within the same time frame.

## Background

The exogenous application of gibberellic acid-3 (GA_3_) during key phases of fruit development is critical for seedless grape varieties, since it promotes berry enlargement through elongation of pericarp cells [1, 2]. Although these treatments are beneficial in terms of meeting the market standards at expected fruit caliber, they also exacerbate berry drop (also known as shattering) during postharvest [3]. This disorder affects negatively the general quality of the fruit, generating relevant economic losses. Its extent can also increase over time depending on postharvest storage conditions [4].

Preventing decay through the application of systematic measures during the postharvest handling of table grape is critical to achieve the commercialization of healthy clusters [5]. Hence it is important that marketable clusters present a non-senescent aspect with green stems and pedicels attached to the berry. The pedicel has been proposed as a main factor involved in berry drop [6] and several varieties have been described to be more prone to this postharvest issue under GA_3_ treatment conditions. Berry drop in cv. Kyoho is related to an increase in rachis hardness caused by expansion and lignification of this structure as a consequence of GA_3_ treatments [7] . Other commercial cultivars have been reported as susceptible, such as Flame Seedless [5] and Ruby Seedless [3]. Thus, genetic background and the growth regulators applied to certain cultivars, particularly GA_3_, seem to play an important role in postharvest berry drop. However, there are also varieties such as cv: Thompson Seedless which can be considered as non-sensitive to berry drop as long as proper management conditions are achieved: with only three GA doses applied for berry size enlargement spanning at specific fruit developmental stages [4, 6].

Reports have indicated several morphological changes that take place in the rachis and pedicel following GA_3_ treatment [8, 9]. It has been found that GA_3_ induces changes in the pedicel by increasing the area of xylem and pith structures [8]. One study proposed that the loss of pedicel flexibility could be one of the main factors underlying berry drop in cv. Thompson Seedless [10]. Recently, analysis of transcripts related to lignin synthesis on genetic backgrounds with contrasting susceptibility to berry drop was reported, suggesting an enhancement of lignification in response to GA_3_ treatments [11]. In other woody species, an increase in the concentration of bioactive gibberellins (GA) enhanced growth and biomass production [12, 13]. In fact, transgenic lines have been engineered to increase bioactive GA and repress lignin biosynthesis in xylem tissue, resulting in enriched biomass destined to liquid fuel production [14, 15]. Therefore, it is suggested that the expected effects of GA_3_ treatments in vascular tissue could be on cell wall-related processes, which in turn impact on the flexibility of this structure.

Transcriptomic approaches have been very useful to characterize the underlying biology of berry development [16–18] and its responses to gibberellin treatments [19, 20]. These studies have been widely conducted on samples obtained from fruit tissues. In this case, our focus is the study of the responses of pedicel tissue to GA_3_ treatment evaluated through a transcriptomic platform, and how this could contribute to understand a complex trait such as berry drop.

The availability of contrasting phenotypes for postharvest berry drop may serve as a useful reference to understand this phenomenon [11]. L23, a line obtained in the framework of INIA’s table grape breeding program, is a genotype with high susceptibility to berry drop compared to cv. Thompson Seedless, considered as a non-sensitive genotype in this study, under similar agroclimatic and management conditions. By capturing transcriptional variation in response to GA_3_ treatments from two contrasting genotypes for berry drop, valuable information can be extracted to unveil the underlying differential response through the identification of differentially expressed genes. The objective of this study was to characterize the early phenotypic and transcriptional responses to GA_3_ application of two genotypes that show contrasting susceptibility to berry drop and to identify the main biological processes involved in each condition.

## Results

### Genotype L23 is more susceptible to postharvest berry drop than cv. Thompson seedless under GA_3_ treatment conditions

**Fig. 1:**
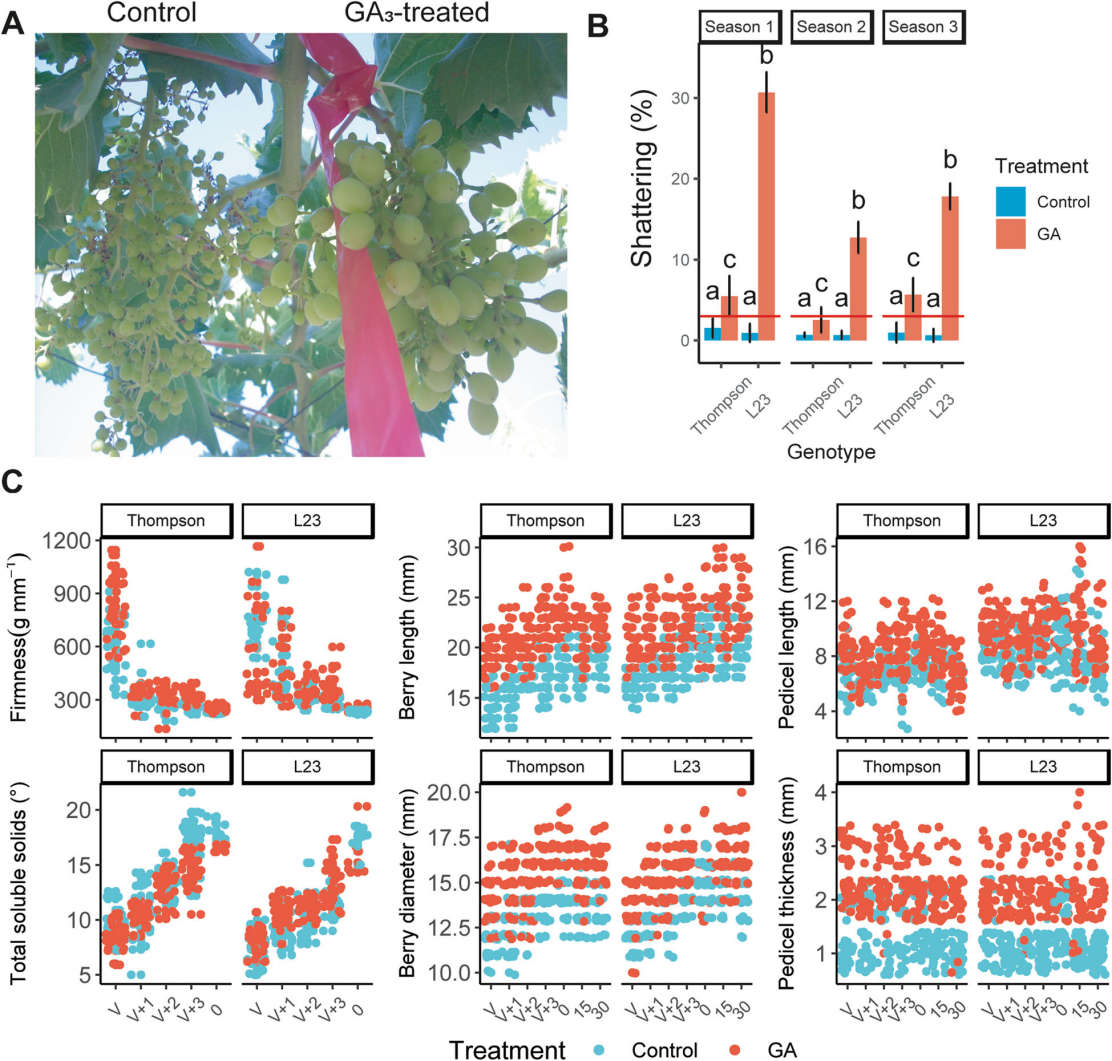
GA_3_ not only increases berry and pedicel dimensions but also exacerbates postharvest berry drop. **a** Effects of GA_3_ application on bunches from cv. Thompson Seedless. The image illustrates the notorious increase on berry size produced by GA_3_ compared to a non-treated cluster of the same vine. **b** L23 exhibits high susceptibility to postharvest berry drop under GA_3_ treatment conditions. Total weight of berries separated from cap stems against total weight of bunches after shaking is reported. Mean and standard deviation are shown on each bar and error bar (replicates for Season 1, Season 2 and Season 3 were 15, 8, 8, respectively, on each point). Red abscissa line in the graph shows 3% berry drop, a recommended tolerance value for high quality table grape exportation. Data were analyzed with ANOVA and Tukey HSD tests to find significant differences between conditions (*p* < 0.05). **c** Pedicel dimensions increase in GA_3_-treated samples. Scatter plots of variables show differences in dimension of berry and pedicel. Total soluble solids and firmness were used as indicators of phenological development. V + *n*: Number of weeks (*n*) passed since veraison (V). The numbers ‘0’, ‘15’, ‘30’ represent the number of days spent in cold storage after harvest time (*n* = 30)

To compare the differential response of L23 and cv. Thompson Seedless in terms of postharvest berry drop, these genotypes were characterized across three seasons following GA_3_-treatment (Fig. 1). The expected effect on berry enlargement after GA_3_ application was confirmed (Fig. 1a). Significant differences in berry drop among conditions by season followed the same trend in all cases; L23 showed higher levels of berry drop than cv. Thompson Seedless but only under GA_3_ treatment conditions (Fig. 1b). In the first season L23 was up to 5.97-fold more susceptible to berry drop. Values for the second and third seasons were 4.11 and 3.11-fold, respectively.

### GA_3_ treatment for berry enlargement promotes pedicel growth and enhances primary cell wall yield

Since GA_3_ treatment is associated to postharvest berry drop, phenotypic variables of treated plants were observed during several developmental stages. Results of the first season are shown in Fig. 1c; two attributes considered as maturity indicators in the study were firmness and total soluble solids of berry samples. As can be seen, the expected effect on berry enlargement was observed under GA_3_ treatment conditions for berry diameter and length. A similar response of the pedicel to GA_3_ treatment was detected, particularly in the thickness of this tissue.

Changes in pedicel dimensions induced by GA_3_ have already been reported [11], and were observed in this study (Additional file 1: Figure S1A). In our study, enlargement of cortex cells and transcript levels of monolignol biosynthesis-related genes (*4CL*, *CAD6* and *CCR1L*) were upregulated by GA_3_ treatment. Transcriptional induction of lignin biosynthesis genes along with increased cell size could be related to the greater stiffness of pedicels as proposed in [10]. To test if the amount of lignin in samples treated with GA_3_ is actually higher in these groups, lignin concentration was determined by the acetyl bromide (AB) method [21]. Successful validation of this method adapted to pedicel tissue was performed using the same reference tissue as in the original research article (Additional file 1: Figure S1B and C).

**Fig. 2:**
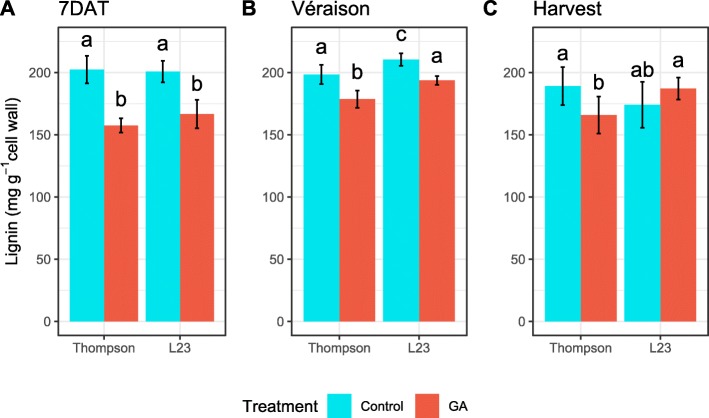
Lignin concentration in pedicels diminishes after GA_3_ treatment. Quantification of soluble lignin by acetyl bromide method of pedicels sampled from three developmental stages. Mean and standard deviation are shown on each bar and error bar, respectively (*n* = 8). ANOVA followed by Tukey tests were performed to detect significant differences between conditions (*p* < 0.05). Stages of evaluation were: **a** Seven days after treatment, **b** veraison and **c** Harvest. Homoscedasticity of data was previously verified by the Levene test, with *p*-values of 0.6583 (**a**), 0.1799 (**b**) and 0.1252 (**c**)

As Fig. 2 shows, lignin concentration in treated groups was unexpectedly lower than in control samples; this difference was more evident at early stages of development, particularly following GA_3_ application (2A). The difference between control and treated conditions in lignin was less accentuated in the véraison stage (2B), and at harvest the observed variation was too great to detect significant differences among conditions (2C). Measurement of samples obtained at a postharvest stage (15 days of cold storage), showed the same trend that at harvest stage (data not shown). Since the method quantifies the amount of soluble lignin obtained from a protein-free cell wall fraction, measurements are relative to the dry matter obtained in each sample.

Considering that pedicel dimensions differed between control and treated groups, changes in dry matter among conditions were recorded. Table 1 reports changes induced by GA_3_ related to dry matter and cell wall accumulation. Dry matter increased in both genotypes; for cell wall yield, which is proportional to the protein-free cell wall fraction obtained from the AB protocol, GA_3_-treated samples showed a significant difference for the L23 genotype.

**Table 1:** Dry matter and cell wall deposition are increased in pedicel samples from GA_3_ treated clusters

Genotype	Treatment	Dry matter (%)	Pedicel fresh weight (mg/pedicel)	Pedicel dry weight (mg/pedicel)	Protein-free cell wall fraction yield (%)^*^
Thompson Seedless	Control	31.0 ± 1.82a (16)	12.6 ± 3.90a (16)	3.86 ± 1.03a (16)	45.4 ± 8.96ab (6)
	GA	34.5 ± 1.52b (16)	73.4 ± 14.8b (16)	25.1 ± 4.27b (16)	52.2 ± 5.18ab (6)
L23	Control	24.5 ± 1.05c (16)	15.2 ± 2.34a (16)	3.71 ± 0.45a (16)	41.8 ± 7.33a (8)
	GA	33.8 ± 1.04b (16)	73.2 ± 12.0b (16)	24.8 ± 4.55b (16)	56.2 ± 5.89b (6)

Mean ± standard deviation is shown in each cell. Number of replicates is presented in parenthesis. Significant differences between conditions in each column are reported as letters (Tukey HSD test: *p* < 0.05)

^*^Starting material corresponded to dried weight of pedicels (~ 0.3 g per sample)

In summary, independent of the berry drop (shattering) amount associated to each genotype, the results suggest that size enlargement-related changes in the pedicel induced by GA_3_ treatments could be related to promotion of primary cell wall yield and secondary cell wall modification-related processes.

### Early transcriptional responses of pedicel to GA_3_ are characterized by an enrichment of processes such as cell cycle, photosynthesis, cell wall modification, phenylpropanoid metabolism and xylem development

**Fig. 3:**
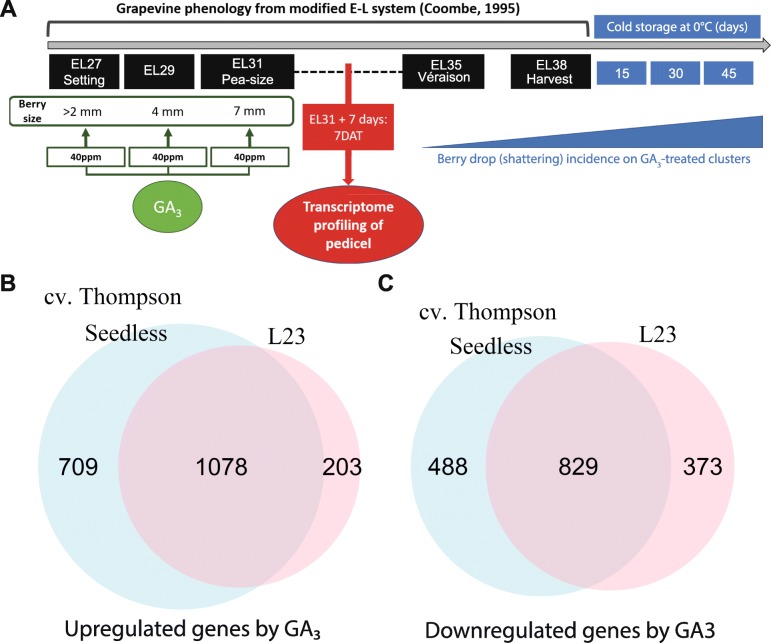
Characterization of the early response to GA_3_-treatment in two backgrounds with different susceptibility to berry drop. **a** The scheme illustrates the rationale behind the timing of pedicel samples since GA_3_-treatment is a major factor involved in berry drop incidence. Phenological-based sampling was critical since the L23 and cv. Thompson Seedless fruit development time frames differ slightly (2–3 weeks). The reference time frame was based on the study of [16]. **b** Upregulated genes in response to GA3 treatments. The number of DE genes was 1787 and 1281 in cv. Thompson seedless and L23, respectively (FDR < 0.05). **c** Repressed genes in response to GA3 treatments. The number of DE genes was 1317 and 1202 in cv. Thompson seedless and L23 s, respectively (FDR < 0.05)

To characterize the early transcriptional responses of pedicel tissue to GA_3_ treatment a transcriptomic profiling of both genotypes to this growth regulator was implemented. Information about treatments and timing of samples is summarized in Fig. 3a (the pipeline followed to perform differential expression analysis is illustrated in Additional file 2: Figure S2).

The number of differentially expressed genes was obtained as indicated in the methods section, after testing relative to a fold-change threshold [22] (Additional file 3: Figure S3). Differential expression analysis revealed a total of 1281 and 1787 upregulated genes for L23 and cv. Thompson Seedless, respectively (Fig. 3b). A total of 1202 and 1317 downregulated genes were detected for L23 and cv. Thompson Seedless (Fig. 3b). This is the first report about differential gene expression in GA_3_ treatments in genotypes with contrasting susceptibility to berry drop (See report for differential expression analysis in Additional file 4: Data S1).

To test if the differentially expressed genes (DEGs) were significantly linked to specific biological processes, gene set enrichment analysis was conducted in gene ontology (GO) annotation terms. Significant GO terms revealed numerous functions associated with enhanced primary cell growth, cell wall modification, histone and chromatin modification, among many others. GO functions of repressed genes were enriched in stress and defense-related processes (See full report in Additional file 5: Data S2).

**Fig. 4:**
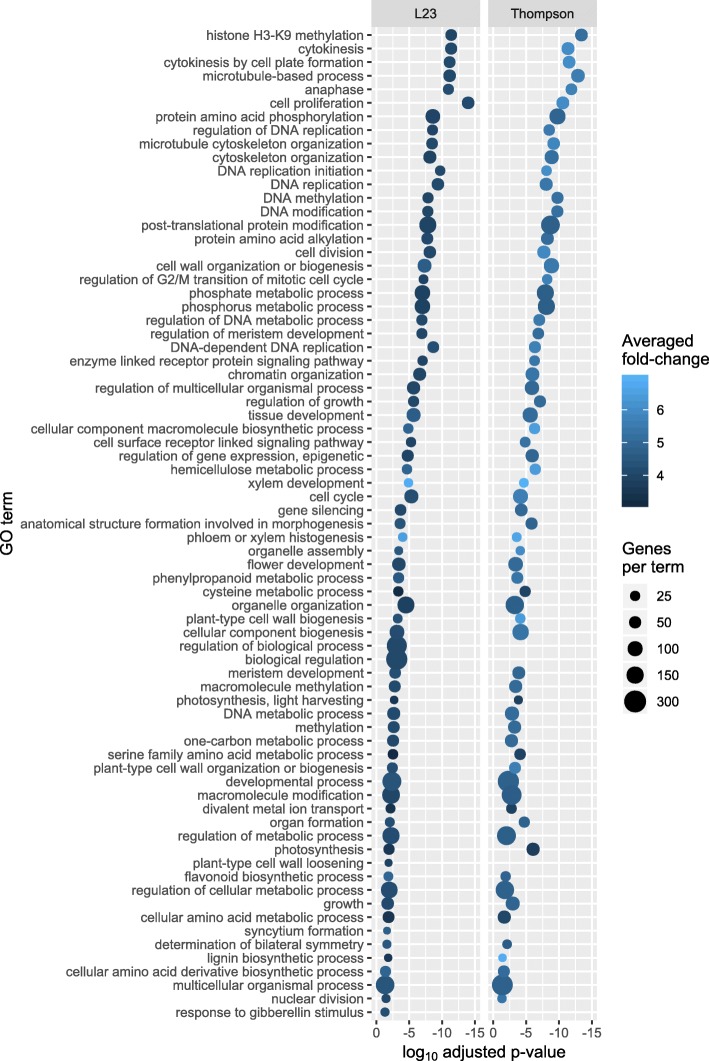
Comparison between genotypes of GO terms enrichment analysis for the upregulated genes by GA_3_. A list of the 73 most prominent and least redundant GO accessions obtained from revigo analysis of L23 (Cutoff value: 0.7) is shown here; this set of GO accessions is compared to the results obtained in cv. Thompson seedless. The log_10_ adjusted p-value is shown here; the size is relative to the number of query items matching the corresponding GO accession obtained from the single enrichment analysis. The fold-change of every DE gene matching each GO accession by genotype was averaged and is shown according to the scale color gradient depicted in the figure. Input of DE upregulated genes was 1281 and 1787 for L23 and cv. Thompson seedless, respectively

To reduce redundancy and detect meaningful information among the GO terms obtained, the method described by Supek et al. [23] was followed. Enriched GO terms for upregulated genes in L23 is shown in Fig. 4. The same analysis conducted on cv. Thompson Seedless can be seen in Additional file 6: Figure S4 (a list with detailed terms and p-adjusted values is given in Additional file 7: Table S1 for L23 and Additional file 8: Table S2 for cv. Thompson Seedless). Several GO terms grouped to cell growth and DNA replication processes. Other relevant functions were related to photosynthesis, xylem development, phenylpropanoid and flavonoid metabolism and cell wall modification processes, among many others.

To corroborate data from transcriptome profiling and validate the results, transcript abundance detected by RNAseq analysis for 18 genes was compared to the measurements obtained by quantitative PCR (qPCR). Genes sampled from RNAseq analysis are given in Additional file 9: Table S3 along with the primers designed for qPCR. Results for the correlation tests between these techniques are given in Additional file 9: Figure S5; qPCR correlated significantly with RNAseq measurements (*p* = 1.368e-13, *R*^*2*^ = 0.80), validating the transcriptomic assay.

In summary, transcriptomic responses to GA_3_ are mainly associated to the regulation of primary cell wall metabolism and cell cycle regulation; which could support primary growth and, by extension, be associated to cell wall fraction yield. Other features such as histone modification, positive regulation of photosynthesis-related genes, flavonoids and phenylpropanoids metabolism, along with xylem development were strongly regulated by GA_3_ in pedicel tissue.

### Genes related to phenylpropanoid, photosynthesis and cell wall pathways were identified in the highly susceptible L23 background as possible biomarkers of berry drop

**Fig. 5:**
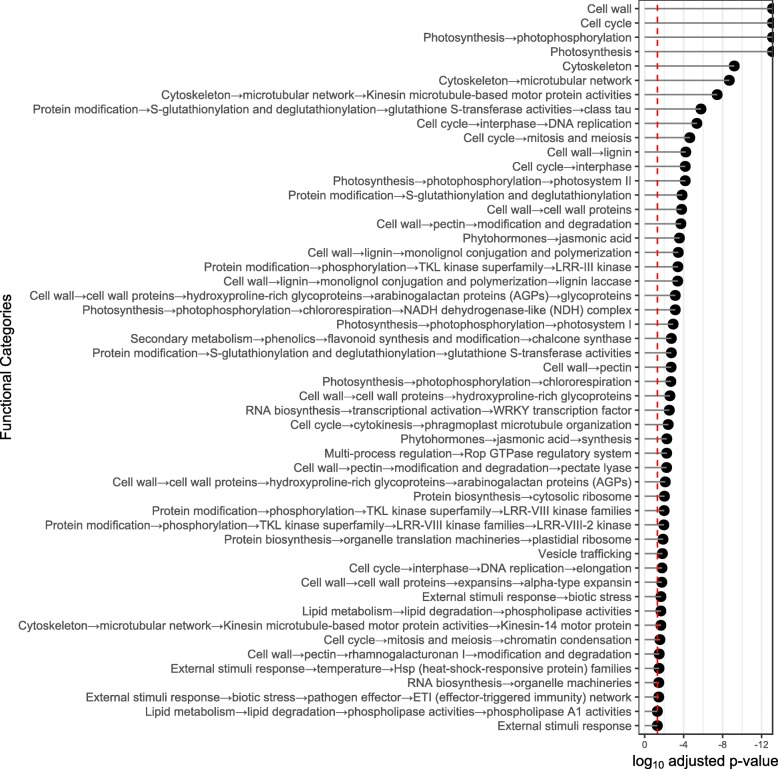
Enriched functional categories by Mapman analysis in L23 genes regulated by GA_3_. All significant categories detected by the Wilcoxon test with p-value adjusted by Benjamini-Yeuketeli are shown on the Cleveland dot plot. Red segmented vertical line depicts threshold significance value (p adjusted value< 0.05). Functional categories were consistent with the results given by the gene ontology analysis and were used as reference in the identification of possible biomarker genes according to the genetic background assessed for the berry drop trait

**Fig. 6:**
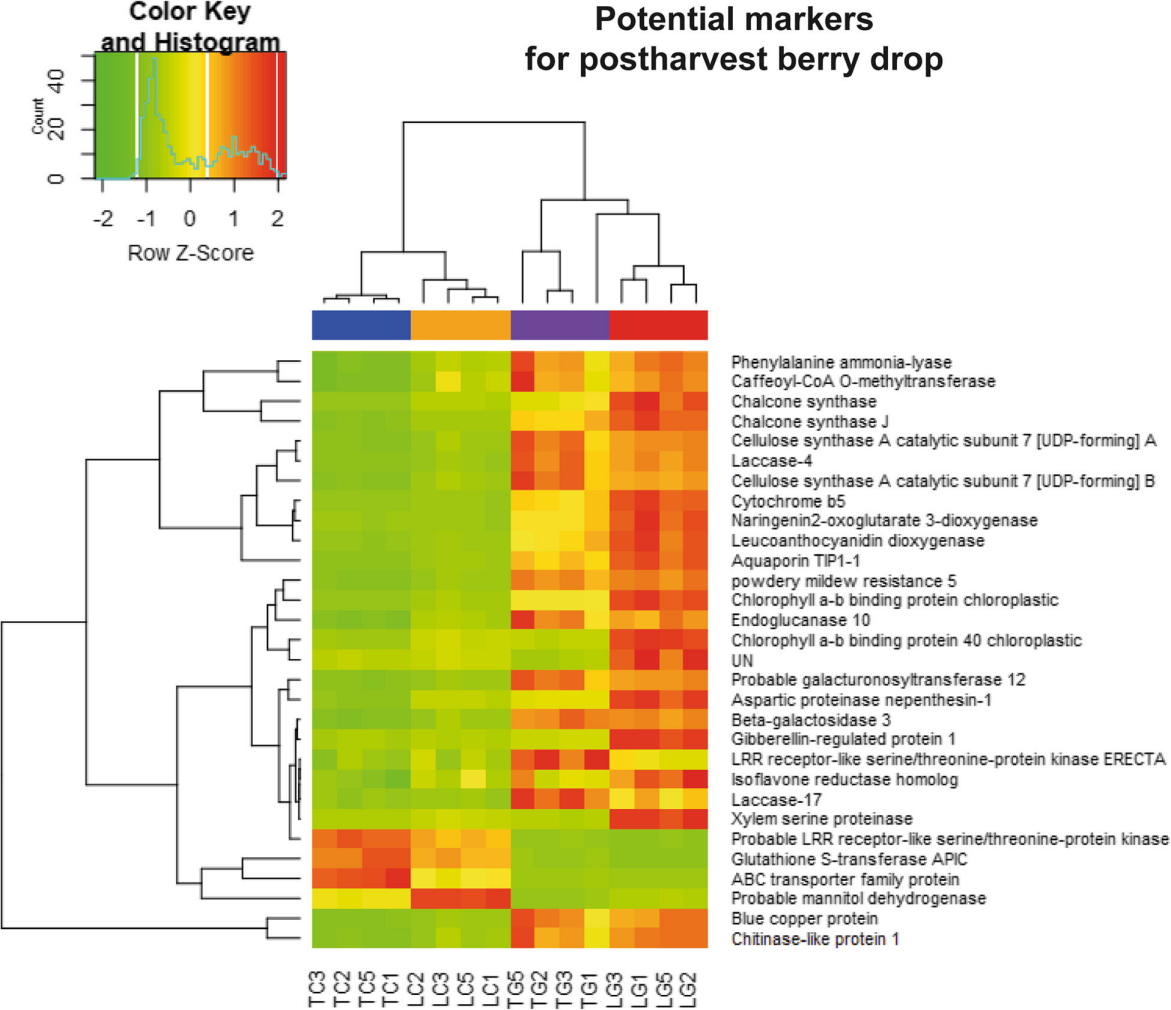
Clustering and heatmap of transcriptomic data from potential candidate genes identified from RNAseq analysis**.** Potential markers were extracted from DE genes. Data corresponds to fragments per kilobase of exon per million reads (FPKM). Below are depicted the libraries from RNAseq analysis (TC: Thompson Untreated, LC: L23 Untreated, TG: Thompson Treated, LG: L23 Treated. Numbers correspond to biological replicates)

To gain insight about the highly regulated genes in the susceptible L23 background, as an approach to associate berry drop and gene regulation, a list of 30 highly regulated genes was obtained from the transcriptomic data. For this, the DEGs grouping under functional categories were identified through the method proposed by [24]; Mapman results can be seen in Additional file 10: Figure S6. Relevant categories were cell wall, along with lignin and some secondary metabolism-related genes. The main reason for the selection of these categories was their high representation as functional categories in terms of associated probability values; all significant categories identified by this method can be seen in Fig. 5. Quantitative transcriptome values of gene expression were fitted to a linear model, considering lowest to highest levels of observed berry drop values (cv. Thompson Seedless untreated, L23 untreated, cv. Thompson Seedless treated, L23 treated) [25]. Regression analysis was performed, and 12 genes were selected (Fig. 6).

### Expression studies of L23 showed predominant regulation of flavonoids, xylem-related genes and secondary cell wall formation

The list of the 12 genes proposed to be studied at several developmental stages comparing the two genotypes is given in Table 2, along with the primers used to measure the abundance of the transcripts by quantitative PCR. Additional file 14 shows that the expression of candidate genes in pedicel samples collected from several stages revealed genes related to secondary cell wall formation such as cellulose synthase subunit-7 (*CESA7*) and endoglucanase (*GUN10)* that are overexpressed under GA_3_ treatment conditions, particularly in the L23 background. Phenylpropanoid-related genes such as phenylalanine ammonia lyase (*PALY*), caffeoyl CoA O-methyltransferase (*CCoAOMT*) and cinnamoyl CoA-reductase (*CCR*) were highly expressed in cv. Thompson Seedless and L23. These are also positively regulated by GA_3_ (*7DAT*) as well as by cold treatment (30 days at 0 °C), most notoriously for the *PALY* gene.

**Table 2:** Primer list of candidate genes to characterize the underlying responses to GA_3_ in pedicel samples from genotypes with contrasting susceptibility to berry drop

Gene id	Description	Ta (C°)	Primer	Sequence (5′ - > 3′)
GSVIVG01009881001	Endoglucanase 10 (*GUN10*)	54	VvGUN10-s	TCG GAC TGC AAA GCT ATC CT
			VvGUN10-a	GCA TTG GGG TCC TTT GAT TT
GSVIVG01031715001	Caffeoyl-CoA O-methyltransferase (*CCoAOMT*)	60	VvCCoAOMT-s	TCA AGC TCA TCA ATG CCA AG
			VvCCoAOMT-a	AGT CAA TCT TGT GGG CAA CC
GSVIVG01023643001	Cellulose synthase A catalytic subunit 7 [UDP-forming] *CESA7*	54	VvCESA7-s	GTC ATT GGT GGT GTG TCA GC
			VvCESA7-a	GGA TAA GGA GGG TGG TCC AT
GSVIVG01032968001	Chalcone synthase (*CHS*)	60	VvCHS-s	CCC GGT GCT GAC TAT CAA CT
			VvCHS-a	AAT CCA GGT GGG TGT CAG AG
GSVIVG01002109001	Probable mannitol dehydrogenase (*MTDH*)	54	VvMTDH-s	TGG TGT TGG GTG TAT GGT TG
			VvMTDH-a	TGT GAT CCC AGC ACA TAG GA
GSVIVG01033677001	Aquaporin TIP1–1 (*TIP11*)	54	VvTIP11-s	AAG AAG GGC AAT TTG GGA AT
			VvTIP11-a	CCT CGT ACA CAA GTC CAG CA
GSVIVG01027584001	Xylem serine proteinase (*XSP1*)	54	VvXSP1-s	TCA GAT ACC GGT TCG GAG AG
			VvXSP1-a	TTG TAT GTG GCG CTG TTG TT
GSVIVG01025703001	Phenylalanine ammonia-lyase (*PALY*)	60	VvPALY-s	AAT TGC AGC CAT TGG AAA AC
			VvPALY-a	GTG TTG CTC AGC ACT TTG GA
GSVIVG01034003001	Laccase 4 (*LAC4*)	54	VvLAC4-s	CTC CCC CAT CGC AGT AGA TA
			VvLAC4-a	TTT GGC TGG GTA CTT TTT GG
GSVIVG01016100001	Aspartic proteinase nepenthesin-1 (*NEP1*)	54	VvNEP1-s	CTC TGA AGG CCG AGT TTC TG
			VvNEP1-a	AGC AAG AGC CAA ACA CAC CT
GSVIVG01001005001	powdery mildew resistance 5 (*PMR5*)	54	VvPMR5-s	GGC ATG ATT CAC TGG GTT CT
			VvPMR5-a	GCT TCC ACC TCC ATT TCT CA
GSVIVG01029789001	Chlorophyll a-b binding protein chloroplastic (*CB12*)	54	VvCB12-s	CCG GTG ACT ATG GCT TTG AT
			VvCB12-a	AGG AGT TGG GTT CCA AGG AG
GSVIVG01011810001	Probable fructose-bisphosphate aldolase 3 chloroplastic (*ALFC3*)	54	VvAFLC3-s	GAT GGG GAT CAC CCA ATT GAT
			VvAFLC3-a	ATT TGG CGA TGG TCT CTG GA

Genes of interest were selected to generate a group of markers to study its variation along several stages of development in genotypes with contrasting berry drop phenotypes. Gene name and description comes from basic local alignment using the Swissprot database

Flavonoid 3′-hydroxylase (*F3H*), an enzyme belonging to the flavonoid biosynthesis pathway, is highly expressed in the L23 genotype and upregulated by GA_3_. The expression pattern of this gene in the susceptible genotype is strikingly different from cv. Thompson. Some laccases, also differentially expressed, such as Laccase-4 (*LAC4*) and Laccase-17 (*LAC17*) may exert an important role in lignin condensation, although their expression was relatively low. Other genes such as Nephentesin-1 (*NEP1*), Xylem serine proteinase (*XSP1*) and Chlorophyll a − b binding protein 40 chloroplastic (*CB12*) had higher basal levels in the L23 background than in cv. Thompson Seedless and were regulated positively by GA_3_. These sets of genes responsive to GA_3_ showed distinctive expression patterns between genotypes and treatments across several stages of development.

## Discussion

Gibberellins are the most used growth regulators in table grape production; particularly in seedless varieties, in which the endogenous generation of the hormone is reduced to basal levels and so the application of GA_3_ is mandatory to obtain berries of marketable size [26]. However, there is also a detrimental effect of GA_3_, which increases postharvest berry drop, a problem well known for a long time for some cultivars such as Thompson Seedless [6]. Some authors have proposed that the rachis and pedicel could be one of the main structures influencing berry drop [9–11]. But scarce information exists about the transcriptional response of this tissue to GA_3_ treatments.

This is the first study to characterize the transcriptomic response of table grape pedicel to GA_3_ in two genetic backgrounds with contrasting susceptibility to berry drop. The main findings were: (i) GA_3_ treatments induced major phenotypical changes in the pedicel, by increasing its size and modifying cell-wall properties; (ii) accumulation of lignin was negatively affected by an early response of cell wall augmentation, resulting in an initial dilution effect with lower concentration of the polymer. As a consequence, lignin concentration reached similar values for both treated and untreated groups at advanced stages such as harvest time; (iii) transcriptome profiling of samples after treatment provided cues on the main biological processes upregulated by GA_3_, which can be summarized as promotion of vegetative growth through positive regulation of cell growth, cell wall modification, xylem development, phenylpropanoid and flavonoid metabolism, along with downregulation of stress-related responses; and (iv) core responses are similar between the genotypes, one more tolerant and one more susceptible to postharvest berry drop, although there are a number of DEGs that are specific to each one. Genes which showed contrasting expression patterns were identified and studied at several stages; this set of genes, or some of them, could be used potentially as indicators or biomarkers of postharvest berry drop. However, to obtain robust indicators it is necessary to generate information in the context of segregating populations to understand the underlying genetics of the shattering trait and subtraits. With this information, comprehensive models can be inferred which could serve to predict the occurrence of this trait in wider genetic backgrounds.

Berry drop in seedless varieties has been linked to growth regulator treatments, most of them correlating with the application of the gibberellic acid-3 (GA_3_) isoform for berry size enlargement [3, 5, 6, 27, 28]. Researchers have reported the effects of this growth regulator on several tissues and conditions. In pedicel tissue, positive regulation by GA_3_ on lignin monomers biosynthesis, cell expansion and xylem development has been detected [8, 11, 28]. The present study supports these findings in pedicel with the identification of upregulated genes with significant annotations for these processes. However, although gene expression data could suggest a possible upregulation of lignin deposition in this tissue, our results showed scarce alteration of lignin quantity after GA_3_ treatment; as a matter of fact, its concentration was diluted in a cell wall matrix which was augmented in response to this plant growth regulator. Polymerization of lignin is a process which seems to be finely regulated by the cellular surroundings [29–31] and could be highly dependent of the analyzed tissue. For instance, a recent study showed that lignin deposition in berry skin is in fact diminished by GA_3_, along with the activities of enzymes which synthetize several lignin monomers [32]. This would suggest that pedicel stiffness is probably more related to a strong cell wall synthesis response elicited by GA_3_ than to a differential response on lignin deposition, which was proposed elsewhere for the contrasting genotypes studied here [11]. However, some studies have shown that pedicel stiffness is not always the expected phenotypic response to GA_3_. For instance, in cv. Italia the berry detachment force (an indicator of pedicel strength) is unaffected by GA_3_ [33]. In summary, berry drop, and the phenotypes associated to GA_3_, could have a wide arrange of responses depending on the genetic background assessed and the tissue analyzed, even under similar agroclimatic conditions. Besides pedicel stiffening, other factors would have taken into account to predict berry drop under postharvest conditions such as brush strength, varietal response to GA_3_ and other relevant postharvest conditions.

Measurement of lignin was coherent with other techniques performed on similar tissues. For example, lignin content in grape stalks has been estimated to be 17.4% by the Klason method (corrected previously by tannin, polyphenols and other substances) [34]. In our study, the range for reported lignin content was 15.7–20.4%. We observed that this diminished content at early stages of GA_3_ application could be explained by a strong response in primary cell wall synthesis that sustains the growth of cell components through creep processes [35]; however, to prove such statement other analyses must be conducted to measure the differences among the different components of the lignocellulosic matrix. This could be an interesting and challenging task to perform in this woody tissue. Nevertheless, recent methodology that facilitates the analysis of cell wall components in other woody species through non-destructive protocols could be critical to correlate cell wall changes with transcriptional data [36] and remains an interesting topic to be addressed in future studies.

A mild but significant difference in cell wall yield was observed between genotypes; however, as mentioned before, the magnitude alone of this parameter will not explain differences in berry drop between backgrounds since the phenomenon could be far more complex and other factors could be involved. The transcriptional profiles of a set of genes, which were mostly involved in primary growth, could correlate with the observed phenotypic changes elicited by GA_3_. The gene with the most different expression profile between genotypes was Cellulose synthase subunit-7 (*CESA7*). which has been described as a gene involved in secondary cell wall synthesis [37]. In this study, this gene was highly expressed in the pedicel of L23 background along with several genes encoding other CESA-related subunits (GSVIVG01023643001, GSVIVG01021248001, GSVIVG01028402001, GSVIVG01033278001; see Additional file 3 for more detailed results). Further analyses will be required to evaluate the specific effects of these genes on cell wall synthesis and its interactions with GA_3_. It would also be interesting to determine if there are sequence differences in these genes and their regulatory upstream regions between cv. Thompson Seedless and L23 backgrounds, to uncover possible regulation motifs or structural variations associated with the different transcription levels detected by transcriptomics and qPCR methods, along with the measurement of cell wall components to corroborate the effects suggested by transcriptional data .

Responses of seedless berry to GA_3_ treatments are strongly oriented towards hormone crosstalk, as observed in cv. Centennial Seedless, which ultimately orchestrates a general response of cell wall relaxation that promotes berry size enlargement [20]. The pedicel, however, showed more enrichment in ‘effector-related’ responses than in hormone-related pathways in the same time frame of 7 days after GA_3_ treatment (7DAT). Remnants of seed tissues in the berry may play a major role in the response to exogenous hormone applications, and since the pedicel is a vascular and supporting tissue of berry growth, the responses of the latter to GA_3_ applications could be focused on downstream pathways, whereas the former is integrating the external stimuli (in this case, mostly GA_3_) to the signals given by the embryo remnants. Results of the gene set enrichment analysis of the pedicel to GA_3_ (Additional file 5: Data S2) showed positive responses on plant cell wall loosening (GO:0009828) and organization (GO:0071669), among other cell wall-related and cell growth processes, which can be attributed to tissue enlargement similar to what has been described in berry tissue. In contrast, hormone-related terms showed scarce enrichment; although, response to gibberellin (GO:0009739) was detected in positive DEGs. Other responses related to hormones in negative DEGs were related to jasmonic acid (GO:0071395), salicylic acid (GO:0009754, GO:0009863, GO:0071446), ABA (GO:009738) and ethylene (GO:0009723, GO:0009692, GO:0009693). It is worth mentioning that in the present study the response to GA_3_ treatment was only evaluated at 7 days after application. As can be seen in [20], sequencing of samples from earlier stages of evaluation (one and 3 days after treatment) showed that hormone-related responses were richer. Thus, it is important to consider how the response to hormone signaling develops over time. Nevertheless, the aim of this study was to evaluate how different is the response to GA_3_ between two genetic backgrounds with contrasting performance in postharvest berry drop.

Enrichment in processes such as photosynthesis, phenylpropanoids and flavonoids highlights interesting features on how pedicel tissue is regulated by GA_3_. Although this study did not include measurements of photosynthetic variables, transcriptomics showed that many photosynthesis-related genes were upregulated by GA_3_ treatment. KEGG analysis based on KEGG Orthologs showed several genes involved in photosynthesis reactions (Additional file 11: Figure S7A). Non-foliar photosynthesis has been discussed as an important alternative source of carbon acquisition [38]. The green shoots from annual woody species, known as stalks, diminish CO_2_ efflux through photosynthesis in their chlorenchyma cells [39]. Therefore, it may be suggested that photosynthetic positive responses of pedicels to GA_3_ could be favorable in the context of sustaining general growth, although the extent of CO_2_ assimilation and its contribution to pedicel and berry growth remains to be further analyzed. The phenylpropanoids pathway showed positive regulation of genes related to the synthesis of these metabolites, except for those involved in the final steps of monolignol biosynthesis (Additional file 11: Figure S7B). These results suggest that phenylpropanoids are upregulated towards the flavonoid pathway in response to GA_3_ (Additional file 11: Figure S7C). Polyphenolic compounds derived from plant secondary metabolism, such as phenylpropanoids and flavonoids, include a wide spectrum of structurally and functionally rich compounds, to which a high antioxidant capacity has been attributed [40]. The role of these compounds in the pedicel and its interaction with GA_3_ seems to be related to a general vegetative response, and further metabolomics analysis could help to identify the variety of secondary metabolites present in this tissue. The abundance of these metabolites is suggested to be higher in treated samples, since polyphenolic compounds interfere with RNA extraction as was observed in this study prior to the optimization of extraction methods for pedicels (data not shown).

Open questions remain about the long-term response of GA_3_-treated tissues, such as the influence of cold treatment during storage conditions and their gene activity, which have not yet been addressed under postharvest conditions. Besides, even though a genotype-dependent factor has been suggested in berry drop, there are no genetic studies linking traits such as berry drop in segregating populations, due to the complexity of phenotyping this disorder. Several efforts have focused on increasing the precision of measurement of berry drop [41]. Hopefully, more studies will help to understand the genetic factors and conditions determining berry drop in table grapes.

## Conclusions

This is the first study to investigate the effects of GA_3_ treatment on the pedicel transcriptomic response of genotypes with different susceptibility to postharvest berry drop. This phenomenon in table grape is related to GA_3_ treatments, which increase pedicel dimensions due to promotion of cell wall and phenylpropanoid metabolism. This is consistent with the differentially expressed genes and gene families found here by a transcriptomic approach. Molecular responses to GA_3_ treatment varied according to the genetic background: genes involved in cell wall synthesis, phenylpropanoid, flavonoid and laccase-related processes were highly upregulated in the L23 susceptible background, which showed a higher incidence of berry drop compared to cv. Thompson Seedless. Positive regulation of these genes could partially explain the differences observed in cell wall yield-related results. Moreover, a strong response of photosynthesis-related genes was detected by gene ontology analysis, suggesting a possible role of the pedicel in non-foliar photosynthesis for both genotypes.

Considering that GA_3_ is a widely used growth regulator for table grape production, the differentially expressed genes identified here could be valuable candidate genes for assisted selection in breeding programs. Future analyses will address the changes in cell wall-related components suggested by the transcriptional data gathered here and could be valuable to characterize the dynamics concerning to this plant growth regulator in the pedicel of table grape.

## Methods

### Plant material

This study was performed at La Platina Research Center that belongs to the Instituto de Investigaciones Agropecuarias (INIA), located in Santiago, Chile (33°34′20′′S; 70°37′32′′W; 630 m.a.s.l.), during the 2015–2016, 2016–2017 and 2017–2018 seasons. The plant material (plants of 5–10 years old grown on their own roots, conducted with the Spanish trellis system and managed under standard irrigation, fertilization and pest management programs) of two seedless table grape genotypes: cv. Thompson Seedless and L23 *(‘Line #23’:* F1 from cv. Ruby Seedless x cv. Centennial Seedless) were used in this study. Six individual plants from each genotype were used for qPCR experiments; four biological replicates were used for RNAseq experiments. Each plant had treated (GA_3_) and untreated (Control) clusters to reduce any bias related to individual variation.

Timing of samples was based on the Einchorn-Lorenz phenology system adapted for *Vitis vinifera* L. by Coombe, 1995 [42]. Stage(s) evaluated in each experiment are indicated in each figure.

Regarding the origin of the genotypes assessed in this study, cv. Thompson Seedless (also known as ‘Sultanina’) is a free, ancestral variety, native to west Asia [43]; it was introduced into Chile by the beginning of twentieth century from California, USA. On the other hand, L23 is a segregant belonging to the table grape breeding program of INIA, which was the result of the crossing of cv. Ruby Seedless x Centennial Seedless. Both parents of ‘L23’ are currently free resources originally bred and registered at California Agricultural Experiment Station (University of California) [44] . As with ‘Thompson seedless’, they were introduced into Chile by the mid-twentieth century and kept through vegetative propagation. Cv. Thompson Seedless and L23 are both available from and maintained at INIA grapevine collection.

### Growth regulator treatment

Gibberellic acid (GA_3_, Pro-Gibb 40% Valent Biosciences Chile S.A., Santiago, Chile) treatments of the two genotypes were as follows: (1) for cv. Thompson Seedless, a total of five applications were carried out: 10 ppm GA_3_ for bunch elongation at pre-bloom stage; 15 ppm for thinning at full bloom stage and 200 ppm for berry enlargement, distributed in three doses of 40, 100 and 60 ppm respectively, at EL27, EL29 and EL31 stages, according to Eichhorn-Lorenz phenology system adapted to grapevine by [42]). (2) In the case of L23, clusters were sprayed three times, only for berry enlargement.:Applications of 40 ppm were at EL27, EL29, and EL31. Dosage for cv. Thompson Seedless and L23 was defined in previous assays, optimizing berry enlargement at the lowest possible value of berry drop, as has been described in [11].

### Physiological parameters

The parameters measured in this study were: berry and pedicel diameter (mm), titratable acidity (g L^− 1^ tartaric acid), soluble solids (% w/w g sucrose per 100 g solution), and firmness (g mm^− 1^) of 10 clusters randomly sampled from different vines. For each variable, 30 healthy (e.g. clusters non-affected by any disease such as powdery mildew) and homogenous berries attached with their cap stems (pedicels) were randomly sampled. The diameter of berries and pedicels was measured using a digital caliper. The soluble solid content of fruit was determined by a refractometer (ATC-1E, Atago, Tokyo, Japan), and was periodically measured to follow the fruit development. The data regarding firmness of berries (considering both skin and flesh) was obtained using a firmness tester (Firmtech II, BioWorks, Wamego, KS). Lastly, titratable acidity was determined by measuring the pooled juice of 10 berries per cluster by titration with 0.1 N NaOH (pH 8.2).

As a part of this evaluation, pedicels from clusters of each individual plant were randomly sampled and pooled (300 ~ 500 pedicels). Then, plant material was immediately frozen in liquid nitrogen and stored at − 80 °C for further molecular analyses (e.g. lignin measurement and transcriptional activity-related assays).

### Berry drop

Clusters from each genotype were collected when the average soluble solids content reached 18° on Brix scale (average of ~ 30 berries randomly sampled). For postharvest storage, 10 clusters per treatment (control and GA_3_-treated) were stored immediately after harvest during 15, 30 and 45 days at 0 °C under standard postharvest management conditions (temperature control, gas control, and application of sulphur dioxide to prevent abiotic deterioration factors such as gray mold) [5] and then berry drop was measured. To estimate the percentage of berry drop (shattering), 10 clusters per condition were weighed before and after being shaken for 30 s; total weight of detached fruit was registered. Then, berry drop percentage was calculated as follows BD% = [(DB + SB)/TB] / 100. In this formula, DB was the total weight of dropped berries during storage time at 0 °C, SB was the total weight of dropped berries after shaking and TB represents the total weight of the cluster.

### Determination of lignin

For lignin estimation, quantitative determination by the acetyl-bromide method [21] was followed with slight modifications. Pedicels were dried at 105 °C for 16 h in an oven and cooled in a vacuum desiccator until the next step. Dry samples (0.3 g) were homogenized in 50 mM potassium phosphate buffer (5 mL, pH 7.0) using an Ultra Turrax T25 homogenizer (IKA-Werke GmbH & Co., Staufen, Germany). The solution was centrifuged (1400×g, 5 min) and the pellet was washed by successive stirring and centrifugation as follows: twice with phosphate buffer (pH 7.0; 7 mL), three times with 1% (v/v) Triton X-100 in pH 50 mM potassium phosphate 7.0 buffer (7 mL), and six times with acetone (5 mL). All supernatants were monitored by spectrophotometric measurements at 280 nm to ensure no contamination with protein and UV-absorbing materials in downstream steps. The pellet was dried in an oven (60 °C, 24 h) and kept cool in a vacuum desiccator until further reaction with acetyl bromide. Samples and a blank solution were prepared as follows: dry matter obtained from the previous step (considered as a protein-free cell wall fraction) was placed in a screw cap 15 mL centrifuge tube (20 mg per sample) containing 0.5 mL 25% acetyl bromide (v/v in glacial acetic acid) and incubated at 70 °C for 30 min. After digestion, samples were cooled in an ice bath and mixed with 0.9 mL of 2 M NaOH and 0.1 mL of 5 M hydroxylamine-HCl. Samples and blank solution were solubilized in 8 mL of glacial acetic acid and absorbance of supernatant was measured at 280 nm immediately after centrifugation (1400×g, 5 mins). Dilution in glacial acetic acid was performed to avoid signal saturation. The standard curve was constructed using alkali lignin (Aldrich 37, 096–7) with a reported absorptivity value of 23.03 g^− 1^ L cm^− 1^ (Additional file 1: Figure S1B). Results were expressed as mg lignin g^− 1^ cell wall as appears in the cited protocol [21].

### Dry matter

For the determination of changes in pedicel dry matter accumulation elicited by GA_3_ treatments, samples from different conditions at harvest were measured in an analytical balance. Samples taken from bunches when berries reached an average of 18 °Brix were pooled into a group of 30~60 pedicels and weighed for fresh weight determination (at least 0.3 g fresh weight to avoid technical error). Then replicates were dried at 105 °C in an oven for 16 h and dry weight was measured [21]. Percentage of dry matter and weight of pedicels was determined, considering the number of pedicels weighed in each batch.

### RNA isolation

Total RNA was isolated from 1.0 g frozen tissue using a modified hot borate method [45], following all the indications listed in the cited protocol with the only exception that sample tissue was reduced to a third of the recommended amount and polyvinylpyrrolidone was doubled to avoid phenolic compound contamination before RNA isolation. The quantity and quality of RNA were assessed with Qubit® 2.0 fluorometer (Invitrogen™ by Life Technologies, Singapore). Spectrophotometric determination of A_260/280_ and A_260/230_ ratios and electrophoresis in 1.2% formaldehyde-agarose gels verified the quality and integrity of extracted RNA.

### Library synthesis and sequencing

For the RNAseq experiment, pooled pedicel samples of individual plants were obtained 7 days after the last GA_3_ application (corresponding to EL31 stage according to the Einhoch-Lorenz phenology system [42], plus 7 days—defined in this study as 7DAT). Two levels were used in the treatment factor, control and GA; and two levels were considered in the genotype factor, cv. Thompson Seedless and L23. Four biological replicates were used for each condition, summing to 16 libraries which were synthesized and sequenced.

Prior to library synthesis, total RNA was assessed by fragment analyzer PROSize® 2.0 version 1.3.1.1 (Advanced Analytical Technologies, Inc., Ames, IA, USA). Mean RNA quality in all samples was 7.35 (SD: 0.56), confirming the integrity of extractions. Then 2.5 μg aliquots were used to isolate poly(A) mRNA for preparation of libraries using TruSeq RNA Sample Prep Kit v2, following the manufacturer’s instructions described in the TruSeq RNA Sample Preparation v2 Guide, Part #15026495 Rev. F (Illumina, Inc.). Libraries were sequenced using the HiSeq 4000 platform (Illumina). Libraries were sequenced as paired-end data (2 × 100 bp).

### Pre-processing of reads

Low-quality reads (Phred score Q < 25, nucleotides with undefined base assignment *N* > 1 and read length < 50) were removed using the wrapper tool Trim Galore! [46]. Contamination with Illumina adapters was handled with the same tool, removing sequences matching the adapters.

### Mapping and matrix count extraction

Alignment of clean reads to the *V. vinifera* L. PN40024 12X reference genome [47] was performed using STAR software, considering paired-end data default parameters [48]. Uniquely mapped reads were kept for generation of a count matrix using HTseq [49]. The gene model was extracted from the CRIBI database. Genes reported were based on Genoscope structural annotation. Summarized mapping results are given in Additional file 12: Table S4.

Data from the count matrix of uniquely mapped reads were visualized to confirm major effects expected from the experimental design. Assessment using multidimensional scaling and hierarchical clustering based on counts per million reads showed no anomalies; differences were given mainly by genotype and treatment factors, with low variability of replicates within groups (for experimental details please see Additional file 12 Figure S8).

### Differential expression analysis

Count matrices were analyzed with EdgeR package [50] under R 3.4.4 software. Correction for composition bias in each sample was handled with the CalcNormFactor option. Then limma-voom transformation of data with was performed with the limma package [25]. Since the number of DEGs was relatively high, an additional filter of log_2_-fold-change was established. To keep a low false discovery rate, the method described in [22] was followed, considering a prior log_2_-fold-change of 1. For differential expression analysis considered an adjusted *p*-value (FDR) < 0.05.

### Gene set enrichment analysis and Revigo

Gene set enrichment analysis was conducted on the Agrigo platform v2.0 [51] using hypergeometric distribution of data; *p*-values were adjusted by the Benjamini-Yeukiteli method and significant terms (FDR < 0.05) were analyzed with the Revigo algorithm to reduce redundancy of terms based on semantic relatedness. The cutoff value used was C: 0.7 [23].

### MapMan analysis and KEGG ortholog visualization

Mapping of differentially expressed genes as determined in 2.8 was performed using MapMan software (X4 version). Annotation of *V. vinifera* L. was downloaded from BioMart of the Phytozome v12.1 resource page; amino acid sequences were fed into the Mercator 4 online tool, creating an up-to-date mapping file. The Wilcoxon test with adjusted p-value by the Benjamini-Yeukiteli method was used to detect significant differences among BIN categories provided by MapMan [24].

Visualization of KEGG orthologs was accomplished by the usage of *Pathview* package under R enviroment [52], which is also available as an online utility [53].

### cDNA synthesis

In the case of qPCR assays which required the synthesis of cDNA, the reverse transcription was accomplished in 2 μg of total RNA as template using a MMLV-RT reverse transcriptase with previously primed oligo dT (Promega, Madison, WI), according to the manufacturer instructions. The obtained cDNA was then stored at − 20 °C until further qPCR assays.

### Real-time qPCR assays

Transcript abundance was assessed by qPCR with the LightCycler® 96 system from Roche (Roche Diagnostics, Mannheim, Germany), as described in [54]. Gene-specific primers (Table 2) were designed using the online software Primer3plus [55] and were synthesized by IDT (Integrated DNA Technologies). The qPCR assays were performed on six biological samples in duplicate.

The reference gene was GSVIVG01011810001 – probable fructose-bisphosphate aldolase 3 chloroplastic (AFLC3). This gene was identified based on RNAseq results; all non-differentially expressed genes were filtered and ranked according to the lowest variance and coefficient of variation, considering the 16 libraries. Five genes were selected, and primers were synthetized (Additional file 13: Table S5). AFLC3 gene values were used in downstream calculations related to relative expression.

### Experimental design and statistical analyses

A fully randomized experimental design was used. The two main factors considered were Genotype factor (two levels: cv. Thompson Seedless and L23*)* and Treatment factor (two levels: Control and GA_3_).

Data were previously analyzed with Levene test to verify homogeneity of variance assumption (*p* > 0.05). Then, Analysis of Variance test (ANOVA) and Tukey’s Honest Significance Difference test (Tukey HSD) were conducted to find significant differences between conditions (*p* < 0.05). These analyses were applied to find significant differences on Fig. 1b (analysis was performed on each season, separately. Since that ‘season’ effect could not be fitted to a linear model), Fig. 2, Additional file 14 and Table 1. The number of observations (n) can be found on the corresponding description of each figure.

To extract differential expressed genes from RNAseq experiments, data was contrasted against a negative bimomial data distribution through EdgeR package [50], obtained *p*-values were applied with the Benjamini-Hochberg method to control false discovery ratio (FDR). As was explained before, since the number of differentially expressed genes (DEGs) was high. The method described by [22] on limma package [25] was applied to consider additional log_2_ fold-change (FC) of the treated over control samples between each genotype with a prior log_2_ FC = 1, p-values were corrected according to Benjamini-Hochberg method and a final FDR < 0.05 was considered to identify DEGs. Please refer to Additional file 3 to see results regarding this set of statistical analyses.

For gene set enrichment analysis on gene ontology (GO) terms, hypergeometric distribution of data was assumed, and calculated p-values were applied with Benjamini-Yekuiteli method to control false discovery at a rate of 0.05 (FDR < 0.05). From here, revigo analysis was conducted [23] (with distance between GO terms based on relative similiraty index). Cutoff value was considered 0.7 in cv. Thompson Seedless and 0.5 in L23.

For mapman [24] results, Wilcoxon test was conducted on assigned categories to detect significant differences p-values were corrected by Benjamini-Hochberg method (FDR < 0.05).

Hierarchal clustering method on heatmap figure (Fig. 6) was accomplished by calculating Euclidean distance matrix among genes (rows) and among replicates (columns), the quantitative variable was the fragment per kilobase of exon per million reads (FPKM). This quantity was normalized to Z-score for the purpose of comparing the abundance on each cell generated by this method.

Data were mostly handled and analyzed under R software environment [56], unless indicated otherwise.

## Supplementary information

**Additional file 1: Figure S1.** Validation of AB method [21] for analysis of lignin content in grapevine pedicel. **A** Pedicels increases its fresh and dry matter in response to GA_3_-treatment. Representative images from pedicels sampled of L23 genotype at harvest time (17.4° Brix). Pedicels were cut and imaged before and after being dried at 105 °C by 16 h on oven to illustrate differences between treated and non-treated groups regarding fresh and dried condition. **B** Calibration curve of standard Lignin Alkali. Catalog No. Aldrich 37, 096–7. **C** Preliminary report of soybean seed coat performed on this research compared to the value given in literature (*n* = 16).

**Additional file 2: Figure S2.** Pipeline for RNAseq analysis**.** Scheme followed for extraction of differentially expressed genes and gene ontology analysis from paired-end sequencing Illumina reads. Green hexagons show programs and some relevant features and/or filters considered on each step.

**Additional file 3: Figure S3.** Differentially expressed genes before and after testing relative to a threshold method [22]. Mean-Difference plots are shown on each figure **A** Response to GA_3_ in L23 genotype (FDR < 0.05) **B** Response to GA_3_ in L23 genotype, considering an additional parameter of prior log-fold-change equal to 1 (FDR < 0.05) **C** Response to GA_3_ in cv. Thompson Seedless (FDR < 0.05) **D** Response to GA_3_ in cv. Thompson Seedless, considering an additional parameter of prior log-fold-change equal to 1 (FDR < 0.05). The number of DE genes is specified on the top right section of each graph.

**Additional file 4: Data S1.** Results for differential expression analysis conducted on L23 and cv. Thompson Seedless.

**Additional file 5: Data S2.** Results for single enrichment analysis on GO categories for L23 and cv. Thompson Seedless.

**Additional file 6: Figure S4.** Reduction and visualization of data from gene set enrichment analysis on overexpressed genes from Thompson Seedless. List of significantly enriched GO terms (FDR < 0.05) was used as input for revigo analysis [23]. Distances between terms was based on sim_rel_ index. Cutoff value: 0.5.

**Additional file 7: Table S1.** GO terms obtained from L23 filtered according to revigo method proposed by [23].

**Additional file 8: Table S2.** GO terms obtained from cv. Thompson Seedless filtered according to revigo method proposed by [23].

**Additional file 9: Table S3.** Primer list specifying the genes used to corroborate results given by the transcriptomic approach**.** Eighteen primers were designed to obtain log_2_-fold-change values. The list details sequences for each gene, gene ID and the corresponding description based on BLAST results of the annotated sequence. **Figure S5.** Correlation between RNAseq and qPCR methods on the measurements of transcript abundance. A significant correlation based on Pearson correlation test (*p* = 1.368e-13) was detected, which was assayed on pedicel samples obtained from 7DAT stage. Eighteen genes were analyzed for both genotypes (cv. Thompson Seedless and L23), dots show the directional response considering fold-changes of averaged values of treated samples over control samples.

**Additional file 10: Figure S6.** Mapman figure of DE genes from L23. Mapping of differentially expressed genes from L23 genotype under GA treatment condition is significantly related to promotion of cell wall-related processes. The DE genes from *L23* were visualized through MapMan software. Blue dots show overexpressed genes while red dots account repressed genes by GA treatment. Grey dots correspond to filtered genes with non-differential expression. The templates for displaying genes were taken from Mapman [24].

**Additional file 11: Figure S7.** Prominent pathways observed to be elicited by GA_3_ on L23 genotype susceptible to berry drop. Three KEGG maps based on KEGG orthologs for *Vitis vinifera* L. are presented in this figure: Photosynthesis **A**, phenylpropanoids **B**, flavonoids **C**. Only transcriptional data is shown and is based on the observed log_2_ fold-change ratios of GA_3_ treated over control samples of differentially expressed genes. The templates for displaying pathways were taken from Pathview [52, 53].

**Additional file 12: Table S4.** Summarized results obtained from mapping to reference genome PN40024. **Figure S8.** Effects on transcriptomic profiles were given mainly by genotype and treatment factors. Two unsupervised methods were performed to identify grouping patterns of samples based on the counts per million reads data obtained after trimming step, before the differential expression analysis. **A** Multidimensional scaling plot from the Euclidean distance matrix calculated from count per million reads by library is shown. Each dot is a library (*n* = 16 libraries). **B** Hierarchical clustering of libraries with heatmap of the 500 most variable genes across samples. Genes were ranked according to its variance from log-counts data, the 500 most variable were extracted and hierarchical clustering method was performed according to Euclidean distance matrix calculated from such set. Rows represents genes and columns represents libraries. Orange corresponds to cv. Thompson Seedless and purple corresponds to L23 genotype. On topleft section, color key and a histogram of gene profile expression is shown. Regarding bottom labels for each column: TC (cv. Thompson Seedless-Control), TG (cv. Thompson Seedless-GA), LC (L23-Control), LG (L23-GA), the numbers is the identifier assigned to each biological replicate.

**Additional file 13: Table S5.** Primer list of reference genes assayed on this study to measure transcriptional expression in pedicel.

**Additional file 14:** Gene expression changes in genes proposed as candidate biomarkers reveal strong regulation by GA3 treatment. Mean and standard deviation are shown on each bar and error bar, respectively (*n* = 6). The reference gene used in this study was AFLC3 (GSVIVG01011810001 – possible fructose-bisphosphate aldolase 3 chloroplastic), identified from RNAseq data. Common letters on top of each bar indicate no significant differences between conditions and were identified based on Tukey’s post-hoc test on log2-transformed expression data (*p* < 0.05).

## Data Availability

The datasets used and/or analysed during the current study are available from the corresponding author on reasonable request.

## Abbreviations

°C: Degrees on Celsius scale
*4CL*: 4-coumarate-CoA ligase
7DAT: Seven days after GA_3_-treatment
A_260/230_: Ratio of the absorbance at 260 nm and 230 nm
A_260/280_: Ratio of the absorbance at 260 nm and 280 nm
AB: Acetyl bromide
*AFLC3*: Probable fructose-bisphosphate aldolase 3 chloroplastic
ANOVA: Analysis of variance
*CAD6*: Cinnamyl-alcohol dehydrogenase 6
*CB12*: Chlorophyll a-b binding protein 40 chloroplastic
*CCoAOMT*: Caffeoyl-CoA O-methyltransferase
*CCR*: Cinnamoyl-CoA reductase
*CCR1L*: Cinnamoyl-CoA reductase 1-like
*CESA7*: Cellulose synthase A catalytic subunit 7 [UDP-forming]
*CHS*: Chalcone synthase
CO_2_: Carbon dioxide
DEGs: Differentially expressed genes
EL17: Eichhorn-Lorenz phenological stage 17
*F3H*: Flavonoid 3′-hydroxylase
FC: Fold-change
FDR: False discovery rate
g: Gram
GA: Gibberellic acid
GO: Gene ontology
*GUN10*: Endoglucanase 10
KEGG: Kyoto Encyclopedia of Genes and Genomes
*LAC17*: Laccase-17
*LAC4*: Laccase-4
mg: Milligram
mL: Milliliter
mM: Milimolar
mm: Millimeter
*MTDH*: Probable mannitol dehydrogenase
*NEP1*: Aspartic proteinase nephentesin-1
nm: Nanometer
*PALY*: Phenylalanine ammonia-lyase
*PMR5*: Powdery mildew resistance 5
ppm: Parts per million
qPCR: Quantitative Polymerase Chain Reaction
RNAseq: Ribonucleic acids sequencing (whole transcriptome shotgun sequencing)
Ta: Annealing temperature
*TIP11*: Aquaporin tonoplast intrinsic protein 1–1
Tukey HSD test: Tukey’s Honest Significant Difference test
v/v: Volume of solute to volume of solution ratio
*XSP1*: Xylem serine proteinase 1
