# Supplementary figures and images for: Transcriptomic study of pedicels from GA3-treated table grape genotypes with different susceptibility to berry drop reveals responses elicited in cell wall yield, primary growth and phenylpropanoids synthesis
Source: BMC Plant Biol. 2020 Feb 10;20:66. doi: 10.1186/s12870-020-2260-6 (PMC7011282; doi:10.1186/s12870-020-2260-6)

**A**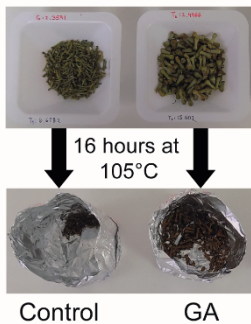**B**

Lignin Alkali (Aldrich 37, 096-7)

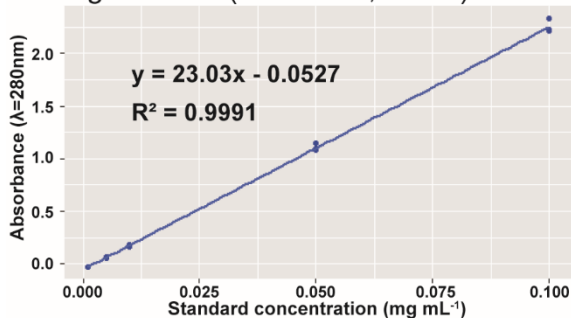**C**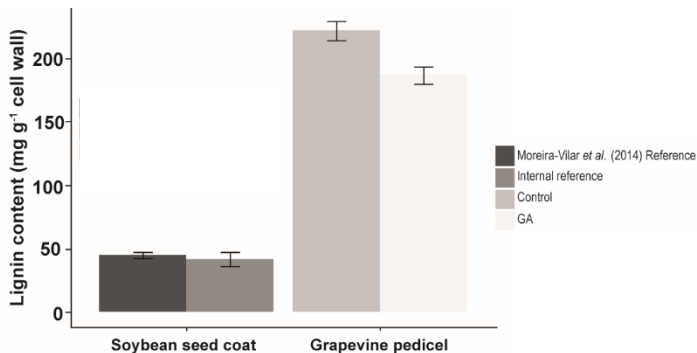

Supplement: Supplementary file 1 — Additional file 1: Figure S1. Validation of AB method [21] for analysis of lignin content in grapevine pedicel. A Pedicels increases its fresh and dry matter in response to GA3-treatment. Representative images from pedicels sampled of L23 genotype at harvest time (17.4° Brix). Pedicels were cut and imaged before and after being dried at 105 °C by 16 h on oven to illustrate differences between treated and non-treated groups regarding fresh and dried condition. B Calibration curve of standard Lignin Alkali. Catalog No. Aldrich 37, 096–7. C Preliminary report of soybean seed coat performed on this research compared to the value given in literature (n = 16). [file 12870_2020_2260_MOESM1_ESM.pdf]

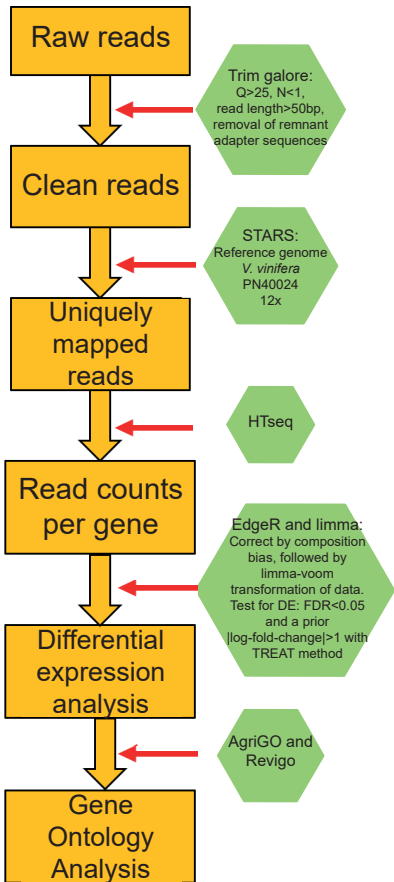

Supplement: Supplementary file 2 — Additional file 2: Figure S2. Pipeline for RNAseq analysis. Scheme followed for extraction of differentially expressed genes and gene ontology analysis from paired-end sequencing Illumina reads. Green hexagons show programs and some relevant features and/or filters considered on each step. [file 12870_2020_2260_MOESM2_ESM.pdf]

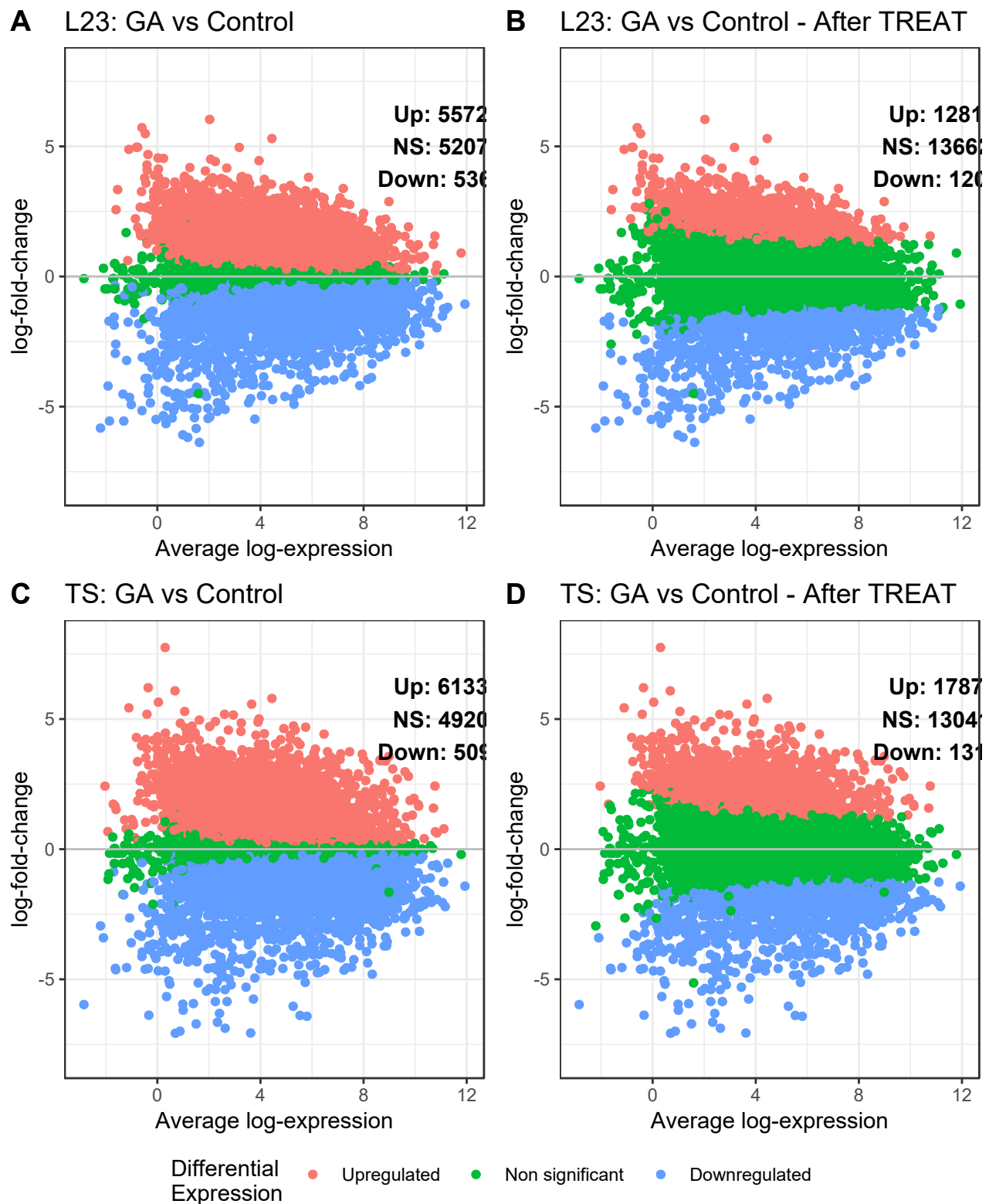

Supplement: Supplementary file 3 — Additional file 3: Figure S3. Differentially expressed genes before and after testing relative to a threshold method [22]. Mean-Difference plots are shown on each figure A Response to GA3 in L23 genotype (FDR < 0.05) B Response to GA3 in L23 genotype, considering an additional parameter of prior log-fold-change equal to 1 (FDR < 0.05) C Response to GA3 in cv. Thompson Seedless (FDR < 0.05) D Response to GA3 in cv. Thompson Seedless, considering an additional parameter of prior log-fold-change equal to 1 (FDR < 0.05). The number of DE genes is specified on the top right section of each graph. [file 12870_2020_2260_MOESM3_ESM.pdf]

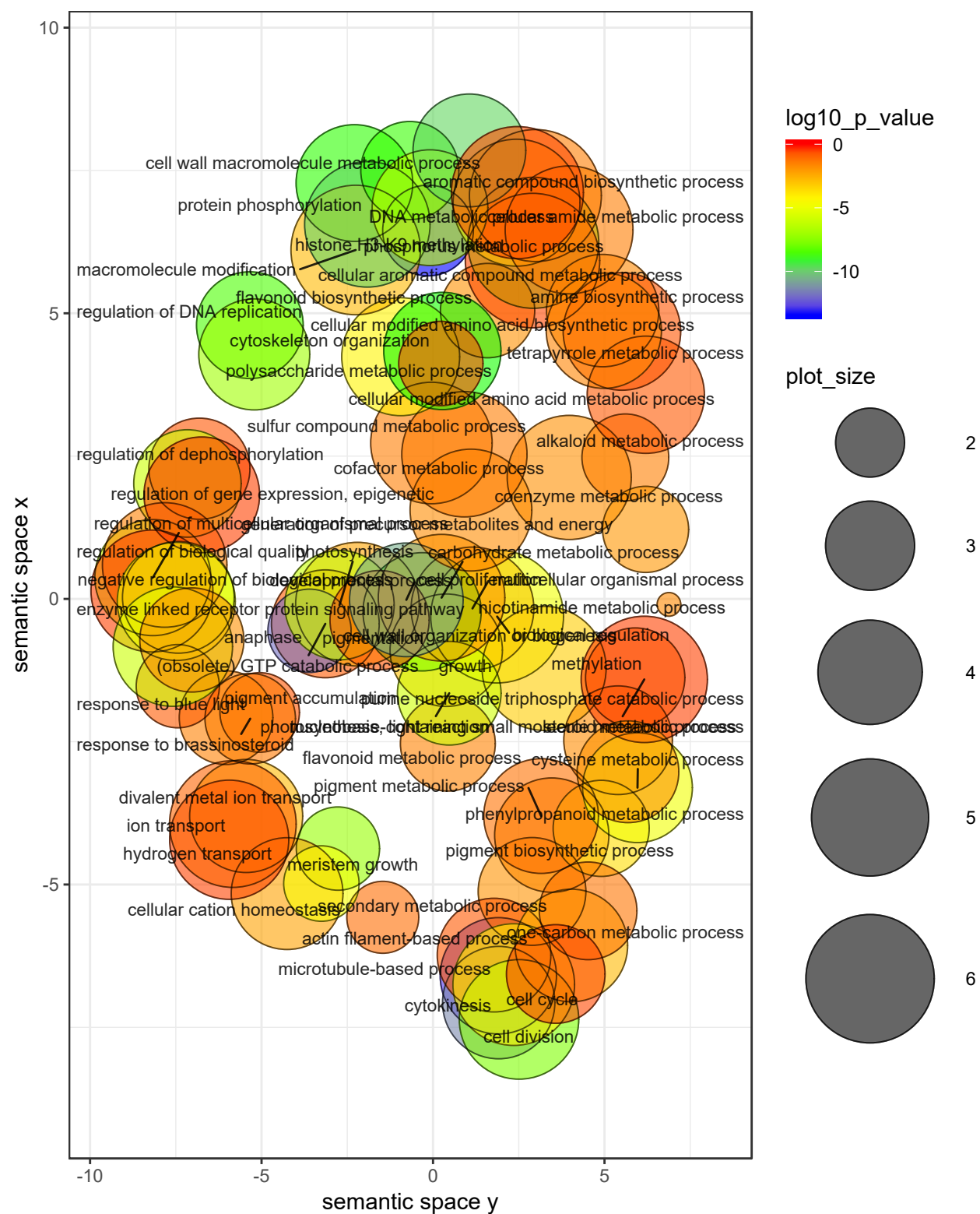

Supplement: Supplementary file 6 — Additional file 6: Figure S4. Reduction and visualization of data from gene set enrichment analysis on overexpressed genes from Thompson Seedless. List of significantly enriched GO terms (FDR < 0.05) was used as input for revigo analysis [23]. Distances between terms was based on simrel index. Cutoff value: 0.5. [file 12870_2020_2260_MOESM6_ESM.pdf]

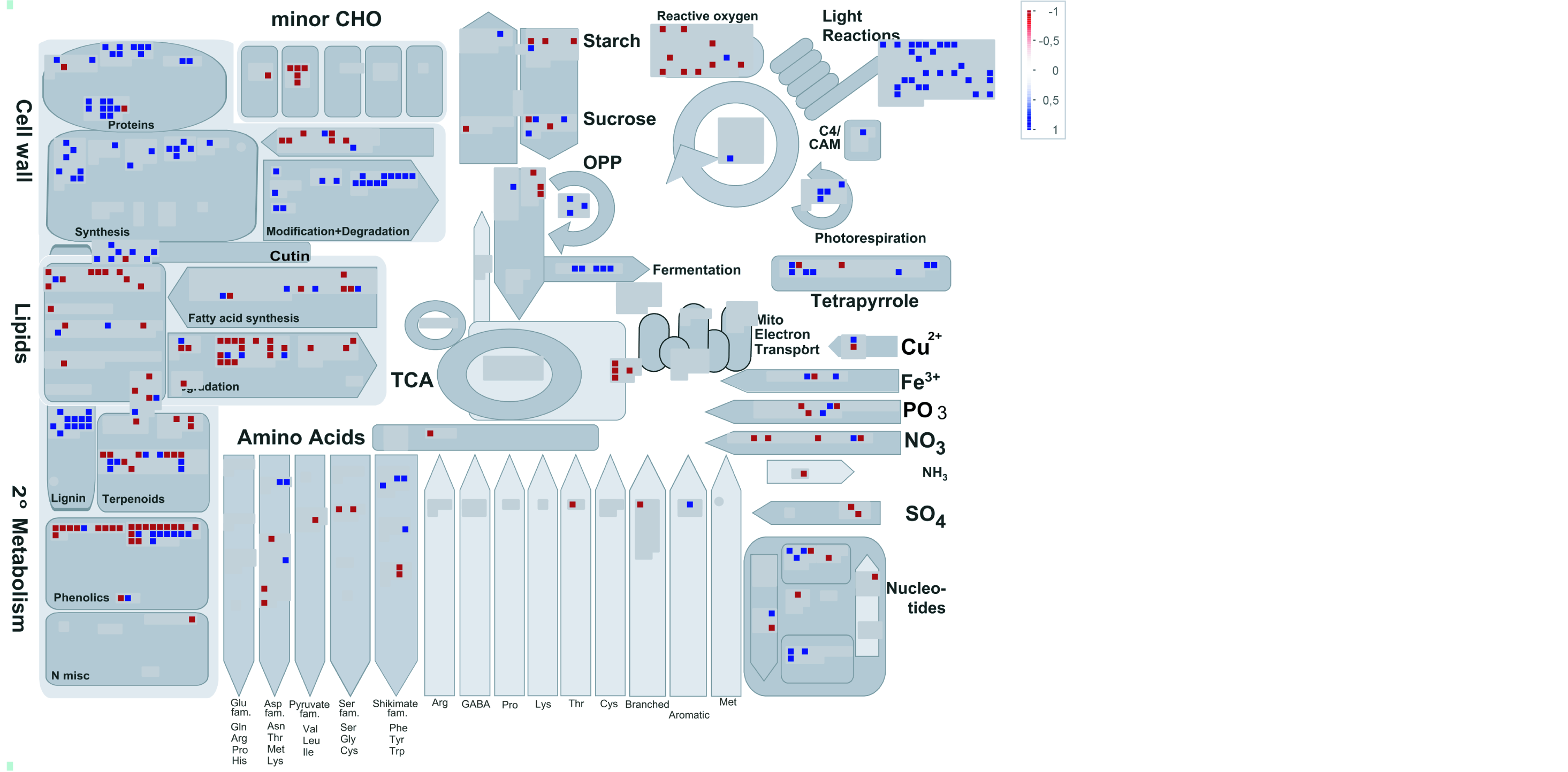

Supplement: Supplementary file 10 — Additional file 10: Figure S6. Mapman figure of DE genes from L23. Mapping of differentially expressed genes from L23 genotype under GA treatment condition is significantly related to promotion of cell wall-related processes. The DE genes from L23 were visualized through MapMan software. Blue dots show overexpressed genes while red dots account repressed genes by GA treatment. Grey dots correspond to filtered genes with non-differential expression. The templates for displaying genes were taken from Mapman [24]. [file 12870_2020_2260_MOESM10_ESM.tif]

## PHOTOSYNTHESIS

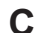

## FLAVONOID BIOSYNTHESIS

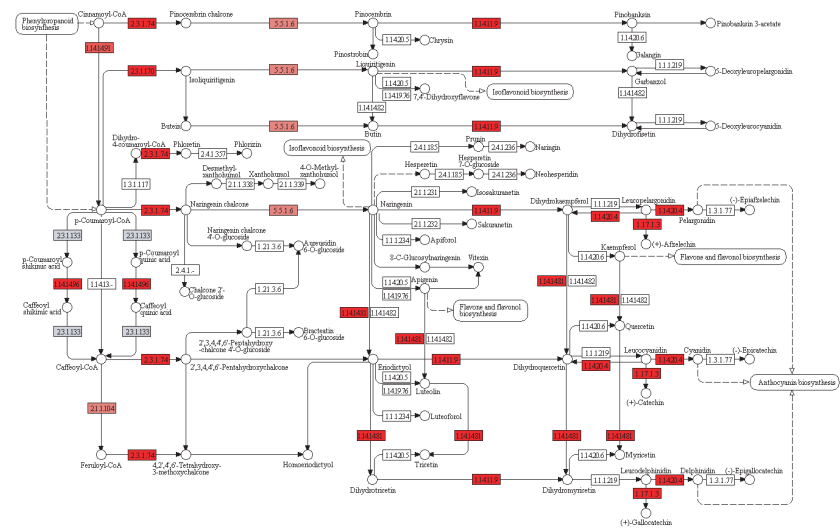

## PHENYLPROPANOID BIOSYNTHESIS

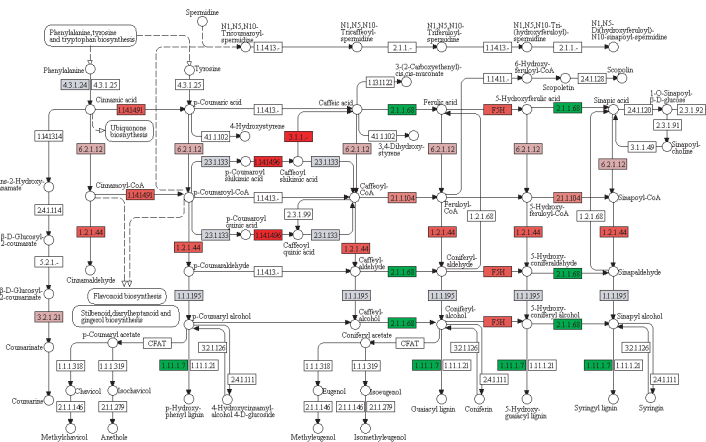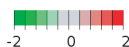

Supplement: Supplementary file 11 — Additional file 11: Figure S7. Prominent pathways observed to be elicited by GA3 on L23 genotype susceptible to berry drop. Three KEGG maps based on KEGG orthologs for Vitis vinifera L. are presented in this figure: Photosynthesis A, phenylpropanoids B, flavonoids C. Only transcriptional data is shown and is based on the observed log2 fold-change ratios of GA3 treated over control samples of differentially expressed genes. The templates for displaying pathways were taken from Pathview [52, 53]. [file 12870_2020_2260_MOESM11_ESM.pdf]
