# Supplementary material for: Transcriptomic study of pedicels from GA3-treated table grape genotypes with different susceptibility to berry drop reveals responses elicited in cell wall yield, primary growth and phenylpropanoids synthesis
Source: BMC Plant Biol. 2020 Feb 10;20:66. doi: 10.1186/s12870-020-2260-6 (PMC7011282; doi:10.1186/s12870-020-2260-6)
Supplement: Supplementary file 8 — Additional file 8: Table S2. GO terms obtained from cv. Thompson Seedless filtered according to revigo method proposed by [23]. [file 12870_2020_2260_MOESM8_ESM.html]

**Enriched terms on cv. Thompson Seedless**

|  | | | |
| GO accession | Term | adj. p-value | Directional response to GA3 treatment |
|  | | | |
| GO:0051567 | histone H3-K9 methylation | 3.9e-14 | Upregulated |
| GO:0007017 | microtubule-based process | 1.3e-13 | Upregulated |
| GO:0051322 | anaphase | 1.3e-12 | Upregulated |
| GO:0000911 | cytokinesis by cell plate formation | 2.9e-12 | Upregulated |
| GO:0000910 | cytokinesis | 4.3e-12 | Upregulated |
| GO:0008283 | cell proliferation | 2.6e-11 | Upregulated |
| GO:0006306 | DNA methylation | 1.7e-10 | Upregulated |
| GO:0006304 | DNA modification | 1.8e-10 | Upregulated |
| GO:0000226 | microtubule cytoskeleton organization | 6.5e-10 | Upregulated |
| GO:0007010 | cytoskeleton organization | 1.3e-09 | Upregulated |
| GO:0071554 | cell wall organization or biogenesis | 1.4e-09 | Upregulated |
| GO:0044036 | cell wall macromolecule metabolic process | 1.7e-09 | Upregulated |
| GO:0043687 | post-translational protein modification | 2.0e-09 | Upregulated |
| GO:0006275 | regulation of DNA replication | 3.1e-09 | Upregulated |
| GO:0010389 | regulation of G2/M transition of mitotic cell cycle | 6.3e-09 | Upregulated |
| GO:0006270 | DNA replication initiation | 7.9e-09 | Upregulated |
| GO:0006793 | phosphorus metabolic process | 8.3e-09 | Upregulated |
| GO:0006260 | DNA replication | 8.9e-09 | Upregulated |
| GO:0051301 | cell division | 2.0e-08 | Upregulated |
| GO:0035266 | meristem growth | 6.6e-08 | Upregulated |
| GO:0040008 | regulation of growth | 7.8e-08 | Upregulated |
| GO:0051052 | regulation of DNA metabolic process | 1.0e-07 | Upregulated |
| GO:0016572 | histone phosphorylation | 3.8e-07 | Upregulated |
| GO:0006261 | DNA-dependent DNA replication | 4.6e-07 | Upregulated |
| GO:0007167 | enzyme linked receptor protein signaling pathway | 5.2e-07 | Upregulated |
| GO:0070589 | cellular component macromolecule biosynthetic process | 5.2e-07 | Upregulated |
| GO:0015979 | photosynthesis | 8.7e-07 | Upregulated |
| GO:0006325 | chromatin organization | 1.1e-06 | Upregulated |
| GO:0040029 | regulation of gene expression, epigenetic | 1.2e-06 | Upregulated |
| GO:0050793 | regulation of developmental process | 1.2e-06 | Upregulated |
| GO:0051239 | regulation of multicellular organismal process | 1.3e-06 | Upregulated |
| GO:0048646 | anatomical structure formation involved in morphogenesis | 1.5e-06 | Upregulated |
| GO:0019684 | photosynthesis, light reaction | 1.5e-06 | Upregulated |
| GO:0006342 | chromatin silencing | 3.1e-06 | Upregulated |
| GO:0006534 | cysteine metabolic process | 1.4e-05 | Upregulated |
| GO:0065007 | biological regulation | 1.5e-05 | Upregulated |
| GO:0010089 | xylem development | 2.2e-05 | Upregulated |
| GO:0005976 | polysaccharide metabolic process | 5.7e-05 | Upregulated |
| GO:0044085 | cellular component biogenesis | 6.9e-05 | Upregulated |
| GO:0007049 | cell cycle | 7.2e-05 | Upregulated |
| GO:0009832 | plant-type cell wall biogenesis | 7.7e-05 | Upregulated |
| GO:0070925 | organelle assembly | 7.8e-05 | Upregulated |
| GO:0009069 | serine family amino acid metabolic process | 8.3e-05 | Upregulated |
| GO:0048449 | floral organ formation | 1.0e-04 | Upregulated |
| GO:0048507 | meristem development | 1.3e-04 | Upregulated |
| GO:0000271 | polysaccharide biosynthetic process | 2.0e-04 | Upregulated |
| GO:0009698 | phenylpropanoid metabolic process | 2.2e-04 | Upregulated |
| GO:0010087 | phloem or xylem histogenesis | 2.7e-04 | Upregulated |
| GO:0050789 | regulation of biological process | 3.7e-04 | Upregulated |
| GO:0043414 | macromolecule methylation | 3.9e-04 | Upregulated |
| GO:0006996 | organelle organization | 5.3e-04 | Upregulated |
| GO:0032259 | methylation | 5.7e-04 | Upregulated |
| GO:0040007 | growth | 1.1e-03 | Upregulated |
| GO:0006259 | DNA metabolic process | 1.4e-03 | Upregulated |
| GO:0043412 | macromolecule modification | 1.6e-03 | Upregulated |
| GO:0006730 | one-carbon metabolic process | 1.7e-03 | Upregulated |
| GO:0070838 | divalent metal ion transport | 1.7e-03 | Upregulated |
| GO:0032502 | developmental process | 5.0e-03 | Upregulated |
| GO:0030003 | cellular cation homeostasis | 5.3e-03 | Upregulated |
| GO:0005975 | carbohydrate metabolic process | 8.3e-03 | Upregulated |
| GO:0019222 | regulation of metabolic process | 9.8e-03 | Upregulated |
| GO:0008652 | cellular amino acid biosynthetic process | 1.0e-02 | Upregulated |
| GO:0006740 | NADPH regeneration | 1.0e-02 | Upregulated |
| GO:0009926 | auxin polar transport | 1.1e-02 | Upregulated |
| GO:0019748 | secondary metabolic process | 1.2e-02 | Upregulated |
| GO:0016128 | phytosteroid metabolic process | 1.2e-02 | Upregulated |
| GO:0006084 | acetyl-CoA metabolic process | 1.2e-02 | Upregulated |
| GO:0080090 | regulation of primary metabolic process | 1.2e-02 | Upregulated |
| GO:0009813 | flavonoid biosynthetic process | 1.3e-02 | Upregulated |
| GO:0006732 | coenzyme metabolic process | 1.4e-02 | Upregulated |
| GO:0007389 | pattern specification process | 1.4e-02 | Upregulated |
| GO:0008202 | steroid metabolic process | 1.7e-02 | Upregulated |
| GO:0065001 | specification of axis polarity | 1.7e-02 | Upregulated |
| GO:0006091 | generation of precursor metabolites and energy | 1.7e-02 | Upregulated |
| GO:0046148 | pigment biosynthetic process | 1.9e-02 | Upregulated |
| GO:0009309 | amine biosynthetic process | 2.0e-02 | Upregulated |
| GO:0022900 | electron transport chain | 2.0e-02 | Upregulated |
| GO:0006769 | nicotinamide metabolic process | 2.0e-02 | Upregulated |
| GO:0022621 | shoot system development | 2.3e-02 | Upregulated |
| GO:0006812 | cation transport | 2.4e-02 | Upregulated |
| GO:0009812 | flavonoid metabolic process | 2.7e-02 | Upregulated |
| GO:0009825 | multidimensional cell growth | 2.9e-02 | Upregulated |
| GO:0065008 | regulation of biological quality | 2.9e-02 | Upregulated |
| GO:0009637 | response to blue light | 3.0e-02 | Upregulated |
| GO:0009820 | alkaloid metabolic process | 3.2e-02 | Upregulated |
| GO:0010817 | regulation of hormone levels | 3.3e-02 | Upregulated |
| GO:0043603 | cellular amide metabolic process | 3.4e-02 | Upregulated |
| GO:0019438 | aromatic compound biosynthetic process | 3.4e-02 | Upregulated |
| GO:0051186 | cofactor metabolic process | 3.6e-02 | Upregulated |
| GO:0009809 | lignin biosynthetic process | 3.9e-02 | Upregulated |
| GO:0010051 | xylem and phloem pattern formation | 4.0e-02 | Upregulated |
| GO:0032501 | multicellular organismal process | 4.1e-02 | Upregulated |
| GO:0006364 | rRNA processing | 4.4e-02 | Upregulated |
| GO:0007349 | cellularization | 4.4e-02 | Upregulated |
| GO:0006636 | unsaturated fatty acid biosynthetic process | 4.4e-02 | Upregulated |
| GO:0000280 | nuclear division | 4.7e-02 | Upregulated |
| GO:0010200 | response to chitin | 2.8e-16 | Downregulated |
| GO:0009620 | response to fungus | 6.1e-16 | Downregulated |
| GO:0006952 | defense response | 2.6e-14 | Downregulated |
| GO:0010033 | response to organic substance | 2.1e-13 | Downregulated |
| GO:0009607 | response to biotic stimulus | 1.0e-12 | Downregulated |
| GO:0002376 | immune system process | 2.4e-12 | Downregulated |
| GO:0006955 | immune response | 2.4e-12 | Downregulated |
| GO:0034976 | response to endoplasmic reticulum stress | 1.2e-11 | Downregulated |
| GO:0006950 | response to stress | 2.6e-11 | Downregulated |
| GO:0012501 | programmed cell death | 6.9e-11 | Downregulated |
| GO:0080134 | regulation of response to stress | 1.5e-10 | Downregulated |
| GO:0050896 | response to stimulus | 1.6e-10 | Downregulated |
| GO:0008219 | cell death | 1.8e-10 | Downregulated |
| GO:0034050 | host programmed cell death induced by symbiont | 2.0e-10 | Downregulated |
| GO:0009626 | plant-type hypersensitive response | 2.0e-10 | Downregulated |
| GO:0009611 | response to wounding | 3.8e-10 | Downregulated |
| GO:0006612 | protein targeting to membrane | 8.3e-10 | Downregulated |
| GO:0009627 | systemic acquired resistance | 1.0e-09 | Downregulated |
| GO:0031407 | oxylipin metabolic process | 1.3e-09 | Downregulated |
| GO:0009694 | jasmonic acid metabolic process | 1.9e-09 | Downregulated |
| GO:0048583 | regulation of response to stimulus | 2.9e-09 | Downregulated |
| GO:0009605 | response to external stimulus | 4.6e-09 | Downregulated |
| GO:0051704 | multi-organism process | 6.7e-09 | Downregulated |
| GO:0033554 | cellular response to stress | 6.7e-09 | Downregulated |
| GO:0023052 | signaling | 5.0e-08 | Downregulated |
| GO:0009695 | jasmonic acid biosynthetic process | 2.1e-07 | Downregulated |
| GO:0009719 | response to endogenous stimulus | 2.2e-07 | Downregulated |
| GO:0051716 | cellular response to stimulus | 3.4e-07 | Downregulated |
| GO:0031408 | oxylipin biosynthetic process | 4.3e-07 | Downregulated |
| GO:0006986 | response to unfolded protein | 4.3e-07 | Downregulated |
| GO:0009408 | response to heat | 5.4e-07 | Downregulated |
| GO:0042538 | hyperosmotic salinity response | 1.0e-06 | Downregulated |
| GO:0045730 | respiratory burst | 2.7e-05 | Downregulated |
| GO:0044283 | small molecule biosynthetic process | 3.4e-05 | Downregulated |
| GO:0009697 | salicylic acid biosynthetic process | 5.1e-05 | Downregulated |
| GO:0009696 | salicylic acid metabolic process | 1.5e-04 | Downregulated |
| GO:0052545 | callose localization | 2.4e-04 | Downregulated |
| GO:0033037 | polysaccharide localization | 2.8e-04 | Downregulated |
| GO:0006865 | amino acid transport | 3.1e-04 | Downregulated |
| GO:0015837 | amine transport | 3.9e-04 | Downregulated |
| GO:0006725 | cellular aromatic compound metabolic process | 6.8e-04 | Downregulated |
| GO:0046394 | carboxylic acid biosynthetic process | 7.4e-04 | Downregulated |
| GO:0032787 | monocarboxylic acid metabolic process | 9.1e-04 | Downregulated |
| GO:0015849 | organic acid transport | 1.0e-03 | Downregulated |
| GO:0009693 | ethylene biosynthetic process | 1.1e-03 | Downregulated |
| GO:0010583 | response to cyclopentenone | 1.6e-03 | Downregulated |
| GO:0050794 | regulation of cellular process | 1.8e-03 | Downregulated |
| GO:0010035 | response to inorganic substance | 2.2e-03 | Downregulated |
| GO:0009404 | toxin metabolic process | 3.7e-03 | Downregulated |
| GO:0009407 | toxin catabolic process | 3.7e-03 | Downregulated |
| GO:0042542 | response to hydrogen peroxide | 3.7e-03 | Downregulated |
| GO:0009595 | detection of biotic stimulus | 7.0e-03 | Downregulated |
| GO:0009644 | response to high light intensity | 7.0e-03 | Downregulated |
| GO:0010150 | leaf senescence | 7.1e-03 | Downregulated |
| GO:0006767 | water-soluble vitamin metabolic process | 9.2e-03 | Downregulated |
| GO:0046482 | para-aminobenzoic acid metabolic process | 1.1e-02 | Downregulated |
| GO:0019438 | aromatic compound biosynthetic process | 1.1e-02 | Downregulated |
| GO:0006082 | organic acid metabolic process | 1.2e-02 | Downregulated |
| GO:0006631 | fatty acid metabolic process | 1.3e-02 | Downregulated |
| GO:0009642 | response to light intensity | 1.4e-02 | Downregulated |
| GO:0006810 | transport | 1.4e-02 | Downregulated |
| GO:0006820 | anion transport | 1.4e-02 | Downregulated |
| GO:0042180 | cellular ketone metabolic process | 1.6e-02 | Downregulated |
| GO:0006457 | protein folding | 1.7e-02 | Downregulated |
| GO:0009628 | response to abiotic stimulus | 1.8e-02 | Downregulated |
| GO:0050789 | regulation of biological process | 2.5e-02 | Downregulated |
| GO:0051179 | localization | 2.6e-02 | Downregulated |
| GO:0065007 | biological regulation | 2.9e-02 | Downregulated |
| GO:0007568 | aging | 2.9e-02 | Downregulated |
| GO:0043900 | regulation of multi-organism process | 3.5e-02 | Downregulated |
| GO:0008152 | metabolic process | 4.1e-02 | Downregulated |
| GO:0010310 | regulation of hydrogen peroxide metabolic process | 4.4e-02 | Downregulated |
| GO:0006979 | response to oxidative stress | 4.6e-02 | Downregulated |
|  | | | |
