# Supplementary material for: Transcriptomic study of pedicels from GA3-treated table grape genotypes with different susceptibility to berry drop reveals responses elicited in cell wall yield, primary growth and phenylpropanoids synthesis
Source: BMC Plant Biol. 2020 Feb 10;20:66. doi: 10.1186/s12870-020-2260-6 (PMC7011282; doi:10.1186/s12870-020-2260-6)
Supplement: Supplementary file 9 — Additional file 9: Table S3. Primer list specifying the genes used to corroborate results given by the transcriptomic approach. Eighteen primers were designed to obtain log2-fold-change values. The list details sequences for each gene, gene ID and the corresponding description based on BLAST results of the annotated sequence. Figure S5. Correlation between RNAseq and qPCR methods on the measurements of transcript abundance. A significant correlation based on Pearson correlation test (p = 1.368e-13) was detected, which was assayed on pedicel samples obtained from 7DAT stage. Eighteen genes were analyzed for both genotypes (cv. Thompson Seedless and L23), dots show the directional response considering fold-changes of averaged values of treated samples over control samples. [file 12870_2020_2260_MOESM9_ESM.pdf]

Table S3.- Primer list specifying the genes used to corroborate results given by the transcriptomic approach**.** Eighteen primers were designed to obtain log_2-fold-change values. The list details sequences for each gene, gene id and the corresponding description based on BLAST result of the annotated sequence.

Gene id	Description	Tm (C°)	Primer	Sequence (5' -> 3')
GSVIVG01010629001	Ethylene responsive factor (ERF)	56.3	VvERF-s	CCT CCT TCA CAT TCC TCC TCA A
		52.5	VvERF-a	CTC CGA TAC CCA TTT ATT ACC TC
GSVIVG01025703001	Phenylalanine ammonia lyase (PAL)	56.7	VvPAL-s	AAG GTC AGG CAA GTT CTG GTA G
		61.8	vvPAL-a	ACC TCT TTG GGC AAA AGG GCC T
GSVIVG01031715001	Caffeoyl-CoA O-methyltransferase (CCOAOMT)	60.2	VvCCOAOMT-s	ATC GGC TAC GAC AAC ACC CTC T
		59.6	VvCCOAOMT-a	CGT CAC CAA CCG GAA GCA TAC A
GSVIVG01028205001	S-adenosylmethionine synthase 5 (METK5)	54.5	VvMETK5-s	CAT CTT CCA TCT CAA CCC ATC A
		59.7	VvMETK5-a	CCC AGC CAC CGT AAG TGT CAA
GSVIVG01032744001	Laccase-12 (LAC12)	54.1	VvLAC12-s	GCA CCA TTT GAC AAT ACT ACC AC
		58.5	VvLAC12-a	CCG TGT CAT TGA AAG CAG GCA A
GSVIVG01033999001	BAHD acyltransferase DCR (DCR)	54.3	VvDCR-s	CCA CAA TTG CTT CAA ACA GTT CT
		60.9	VvDCR-a	TCC GTC TGC GAG TTC CGT GA
GSVIVG01009720001	3-methyladenine DNA glycosidase (JMT)	55.0	VvJMT-s	CTC CAT ACT ATG CAC CAA GTC C
		56.1	VvJMT-a	CCA TCA CCT CCA TCC CAA TCT A
GSVIVG01034174001	Metallothionein-like protein type 2 (MT2)	61.2	VvMT2-s	AGG AAA CTG CGG TTG TGG GTC T
		61.5	VvMT2-a	TGA TGG TCT CAG TGG TGG TGG C
GSVIVG01035962001	Chorismate mutase-2 (CM2)	56.7	VvCM2-s	TGC CTC TTC TTT CCT TCA CTC C
		54.2	VvCM2-a	AAG GTG GTA AAT CAT CTG GGA AA
GSVIVG01026477001	Polygalacturonase (PG)	57.9	VvPG-s	TCG GAG GGC TAT ACG GTA CAT G
		55.9	VvPG-a	AAA GGG TGA AAT TGC TGG TGA G
GSVIVG01016615001	Aquaporin TIP1 (TIP1)	59.6	VvTip1-s	TCT CCC TAT CCT CAG GCG TGT
		57.6	VvTip1-a	GGT GCA ATA ATG CCC AAG TTC C
GSVIVG01033651001	S-adenosylmethionine synthase 3 (METK3)	58.0	VvMETK3-s	TTG CAG AGG CAT TGG GTT TGT T
		57.0	VvMETK3-a	TCC GAT TTC CTC AGG CTT CTT G
GSVIVG01033677001	Aquaporin TIP11 (TIP11)	59.8	VvTIP11-s	AAG AAG GGC AAT TTG GGA AT
		59.9	VvTIP11-a	CCT CGT ACA CAA GTC CAG CA
GSVIVG01028402001	Cellulose synthase A catalytic subunit 7 [UDP-forming] (CESA7)	61.0	VvCESA7-s	AAA GAG AGC GGC TTT CAA GG
		60.1	VvCESA7-a	TCC AGC CAT TTC AAC TTT CC
GSVIVG01032968001	Chalcone synthase (CHSY)	60.1	VvCHSY-s	CCC GGT GCT GAC TAT CAA CT
		60.0	VvCHSY-a	AAT CCA GGT GGG TGT CAG AG
GSVIVG01027584001	Xylem serine proteinase (XSP1)	60.2	VvXSP1-s	TCA GAT ACC GGT TCG GAG AG
		60.2	VvXSP1-a	TTG TAT GTG GCG CTG TTG TT
GSVIVG01002109001	Probable mannitol dehydrogenase (MTDH)	60.1	VvMTDH-s	TGG TGT TGG GTG TAT GGT TG
		60.1	VvMTDH-a	TGT GAT CCC AGC ACA TAG GA
GSVIVG01009881001	Endoglucanase 10 (GUN10)	60.0	VvGUN10-s	TCG GAC TGC AAA GCT ATC CT
		60.0	VvGUN10-a	GGA TTG GGG TCC TTT GAT TT

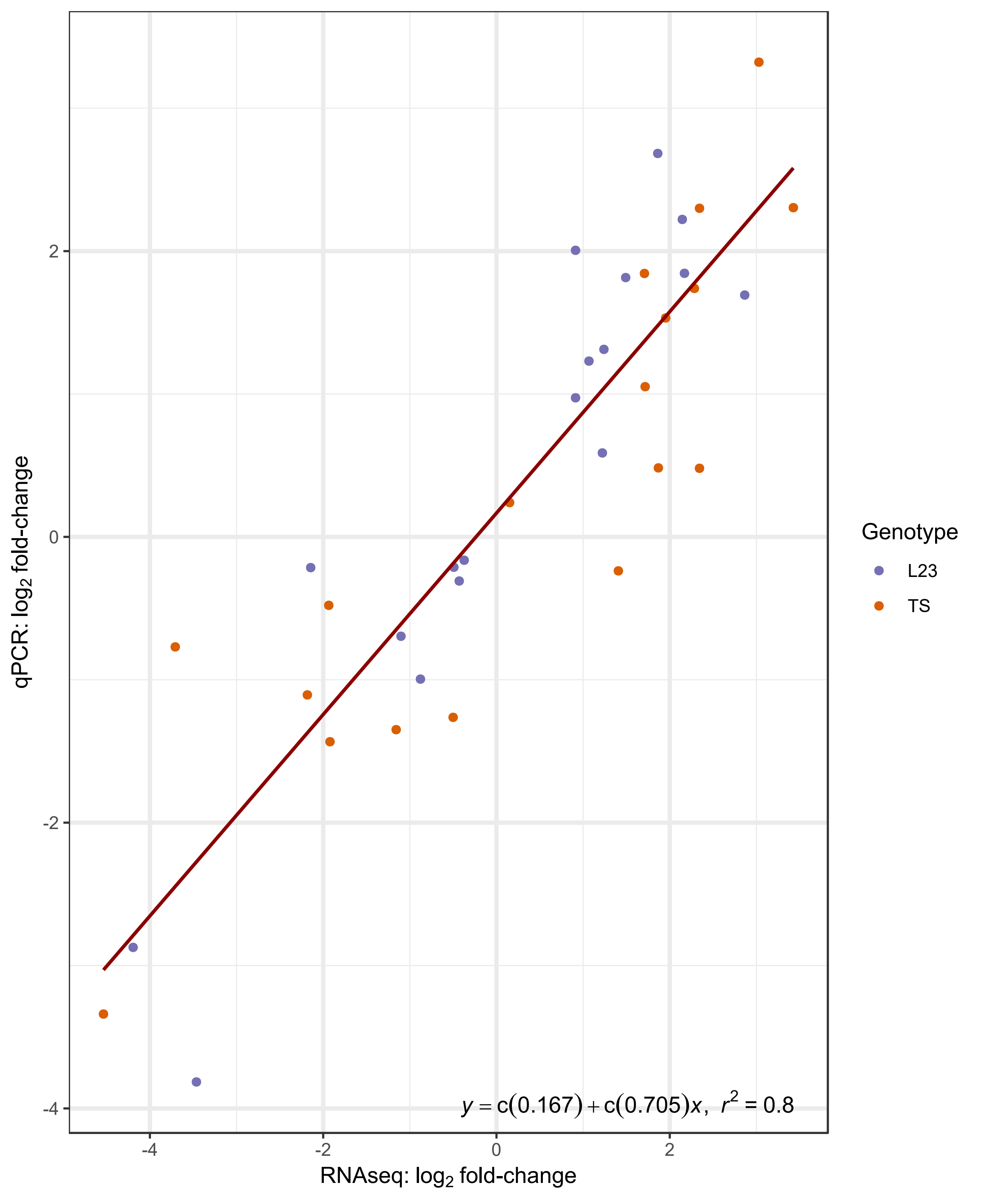

y = c(0.167) + c(0.705)x, r^2 = 0.8
qPCR: log2 fold-change
RNAseq: log2 fold-change
2
0
-2
-4
-4
-2
0
2
Genotype
L23
TS
