# Supplementary material for: Transcriptomic study of pedicels from GA3-treated table grape genotypes with different susceptibility to berry drop reveals responses elicited in cell wall yield, primary growth and phenylpropanoids synthesis
Source: BMC Plant Biol. 2020 Feb 10;20:66. doi: 10.1186/s12870-020-2260-6 (PMC7011282; doi:10.1186/s12870-020-2260-6)
Supplement: Supplementary file 12 — Additional file 12: Table S4. Summarized results obtained from mapping to reference genome PN40024. Figure S8. Effects on transcriptomic profiles were given mainly by genotype and treatment factors. Two unsupervised methods were performed to identify grouping patterns of samples based on the counts per million reads data obtained after trimming step, before the differential expression analysis. A Multidimensional scaling plot from the Euclidean distance matrix calculated from count per million reads by library is shown. Each dot is a library (n = 16 libraries). B Hierarchical clustering of libraries with heatmap of the 500 most variable genes across samples. Genes were ranked according to its variance from log-counts data, the 500 most variable were extracted and hierarchical clustering method was performed according to Euclidean distance matrix calculated from such set. Rows represents genes and columns represents libraries. Orange corresponds to cv. Thompson Seedless and purple corresponds to L23 genotype. On topleft section, color key and a histogram of gene profile expression is shown. Regarding bottom labels for each column: TC (cv. Thompson Seedless-Control), TG (cv. Thompson Seedless-GA), LC (L23-Control), LG (L23-GA), the numbers is the identifier assigned to each biological replicate. [file 12870_2020_2260_MOESM12_ESM.pdf]

Table S4 Mapping results to *V. vinifera* L. reference genome PN40024. Mean and standard deviation is shown on each cell (n=4). Mapping ratio was calculated considering the total reads filtered after trimming step and the uniquely mapped reads obtained from alignment to the reference genome.

		Reads (M)				
**Genotype**	**Treatment**	**Raw**	**After trimming**	**Uniquely mapped**	**Multiple loci**	**Mapping ratio (%)**
cv. Thompson Seedless	GA	21.4 ± 1.4	21.3 ± 1.4	19.8 ± 1.4	0.6 ± 0.04	93.2 ± 1.31
	Control	22.0 ± 1.7	21.6 ± 1.9	19.9 ± 1.7	0.8 ± 0.08	92.1 ± 0.94
L23	GA	24.2 ± 2.1	23.9 ± 2.1	22.7 ± 1.9	0.7 ± 0.06	94.9 ± 1.22
	Control	20.9 ± 3.4	20.9 ± 3.4	19.8 ± 3.3	0.8 ± 0.13	94.8 ± 0.03

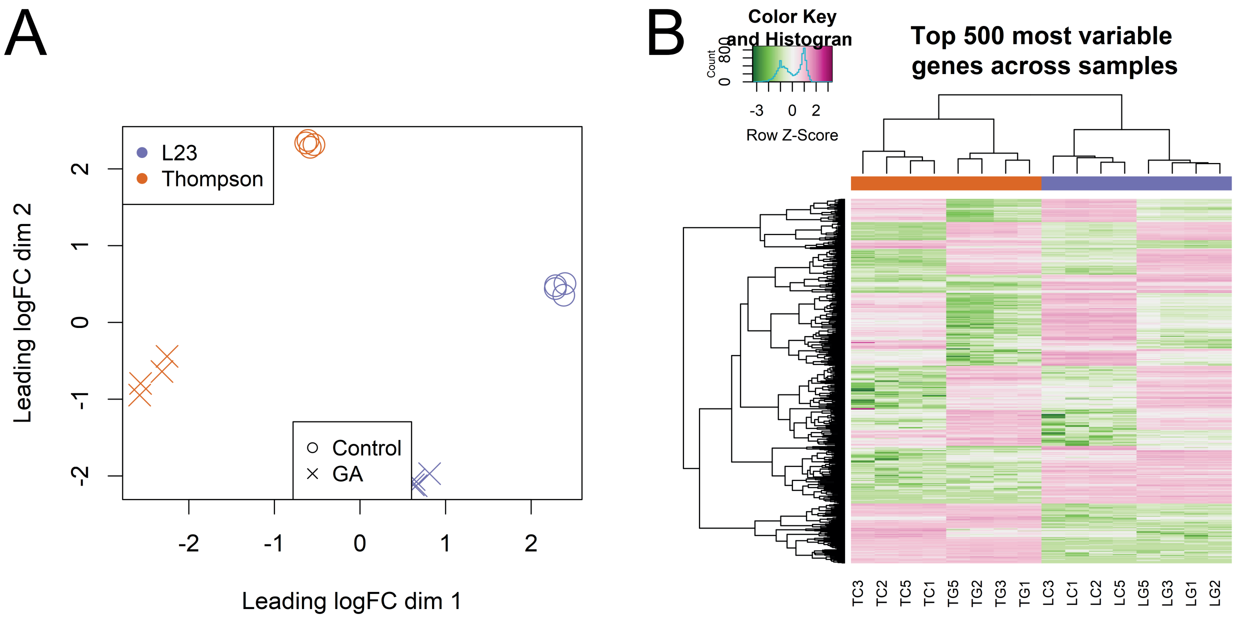
A
L23
Thompson
Control
GA
Leading logFC dim 1
Leading logFC dim 2
-2
-1
0
1
2
B
Color Key
and Histogran
Count
0
800
-3
0
2
Row Z-Score
Top 500 most variable
genes across samples
TC3
TC2
TC5
TC1
TG5
TG2
TG3
TG1
LC3
LC1
LC2
LC5
LG5
LG3
LG1
LG2
