# Supplementary material for: Transcriptomic study of pedicels from GA3-treated table grape genotypes with different susceptibility to berry drop reveals responses elicited in cell wall yield, primary growth and phenylpropanoids synthesis
Source: BMC Plant Biol. 2020 Feb 10;20:66. doi: 10.1186/s12870-020-2260-6 (PMC7011282; doi:10.1186/s12870-020-2260-6)
Supplement: Supplementary file 13 — Additional file 13: Table S5. Primer list of reference genes assayed on this study to measure transcriptional expression in pedicel. [file 12870_2020_2260_MOESM13_ESM.pdf]

Table S5.- Primer list of non-differential expressed genes used as reference transcripts in relative expression measurements.

Gene id	Description	Tm (C°)	Primer	Sequence (5’ -> 3’)	Fragment size (bp)
GSVIVG01011810001	Probable fructose-bisphosphate aldolase 3 chloroplastic (ALFC3)	54.9	AFLC3-s	GAT GGG GAT CAC CCA ATT GAT	167
		56.4	AFLC3-a	ATT TGG CGA TGG TCT CTG GA	
GSVIVG01016795001	60S ribosomal protein L9 (RL9)	52.9	RL9-s	CGA TAT GCT TGA TGG GGT TT	101
		55.4	RL9-a	GAG CAG CAG ATC GTG AAA CA	
GSVIVG01016313001	60S acidic ribosomal protein P0 (RLA0)	56.4	RLA0-s	TCA CCC CTG TGG AGC TTA TC	161
		56.7	RLA0-a	GCT GCT AGG GTT GGG TAT GA	
GSVIVG01015254001	Asparaginyl-tRNA synthetase cytoplasmic 1 (SYNC1)	53.3	SYNC1-s	CCT GTT ATC GTT CAT GAT TAC CC	100
		54.1	SYNC1-a	TTG GTA CAA GAA CAT CCA TAG CA	
GSVIVG01012108001	Coatomer subunit delta (COPD)	56.9	COPD-s	ACT GCT GGC CAT CTG TTT CT	100
		53.5	COPD-a	TCC ATT CCC CAT CAA TTT GC	
