# Supplementary material for: Transcriptomic study of pedicels from GA3-treated table grape genotypes with different susceptibility to berry drop reveals responses elicited in cell wall yield, primary growth and phenylpropanoids synthesis
Source: BMC Plant Biol. 2020 Feb 10;20:66. doi: 10.1186/s12870-020-2260-6 (PMC7011282; doi:10.1186/s12870-020-2260-6)
Supplement: Supplementary file 14 — Additional file 14: Gene expression changes in genes proposed as candidate biomarkers reveal strong regulation by GA3 treatment. Mean and standard deviation are shown on each bar and error bar, respectively (n = 6). The reference gene used in this study was AFLC3 (GSVIVG01011810001 – possible fructose-bisphosphate aldolase 3 chloroplastic), identified from RNAseq data. Common letters on top of each bar indicate no significant differences between conditions and were identified based on Tukey’s post-hoc test on log2-transformed expression data (p < 0.05). [file 12870_2020_2260_MOESM14_ESM.pdf]

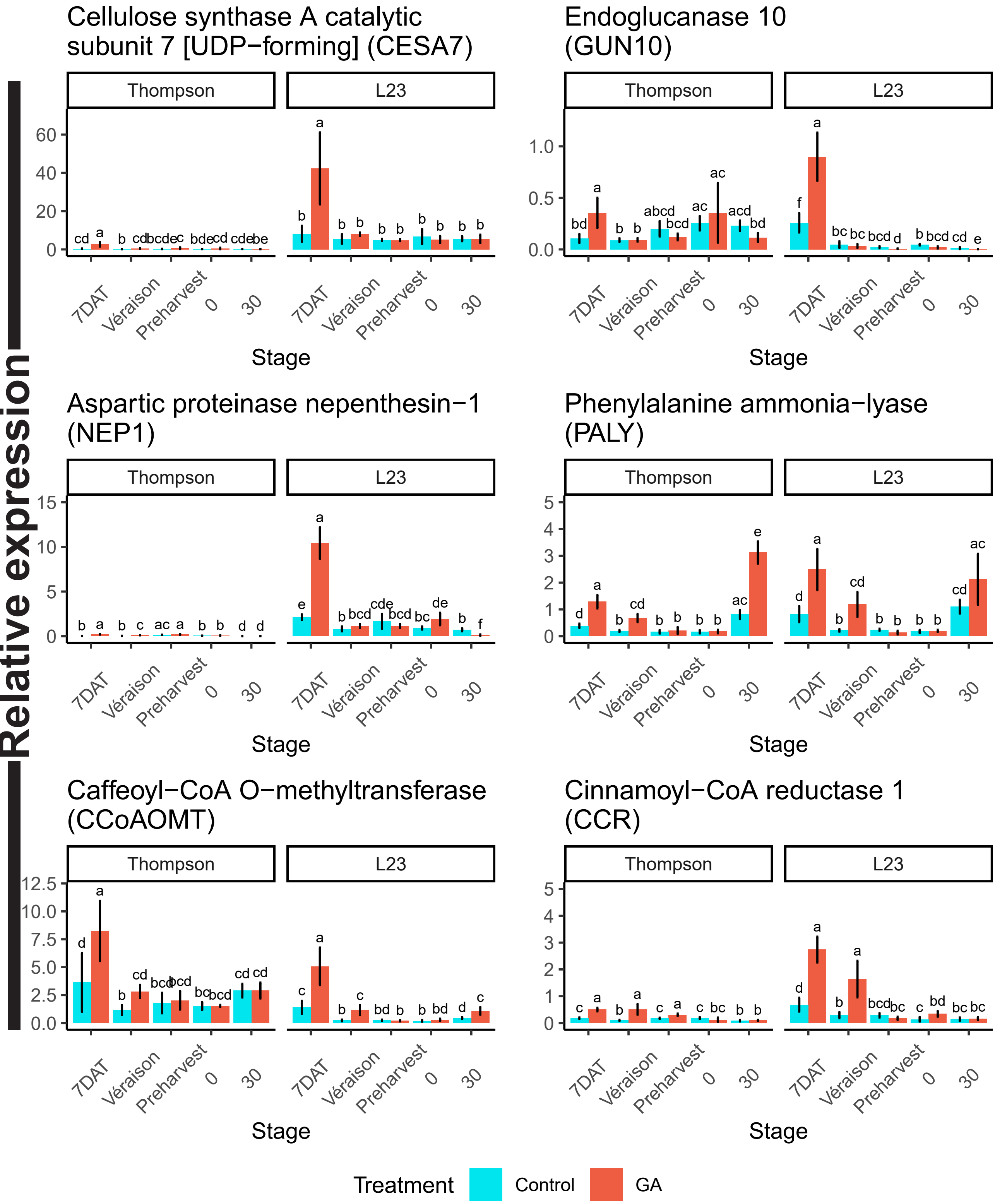
Relative expression
Cellulose synthase A catalytic subunit 7 [UDP−forming] (CESA7)
Endoglucanase 10 (GUN10)
Aspartic proteinase nepenthesin−1 (NEP1)
Phenylalanine ammonia−lyase (PALY)
Caffeoyl−CoA O−methyltransferase (CCoAOMT)
Cinnamoyl−CoA reductase 1 (CCR)
Thompson
L23
7DAT
Véraison
Preharvest
0
30
Stage
Treatment
Control
GA

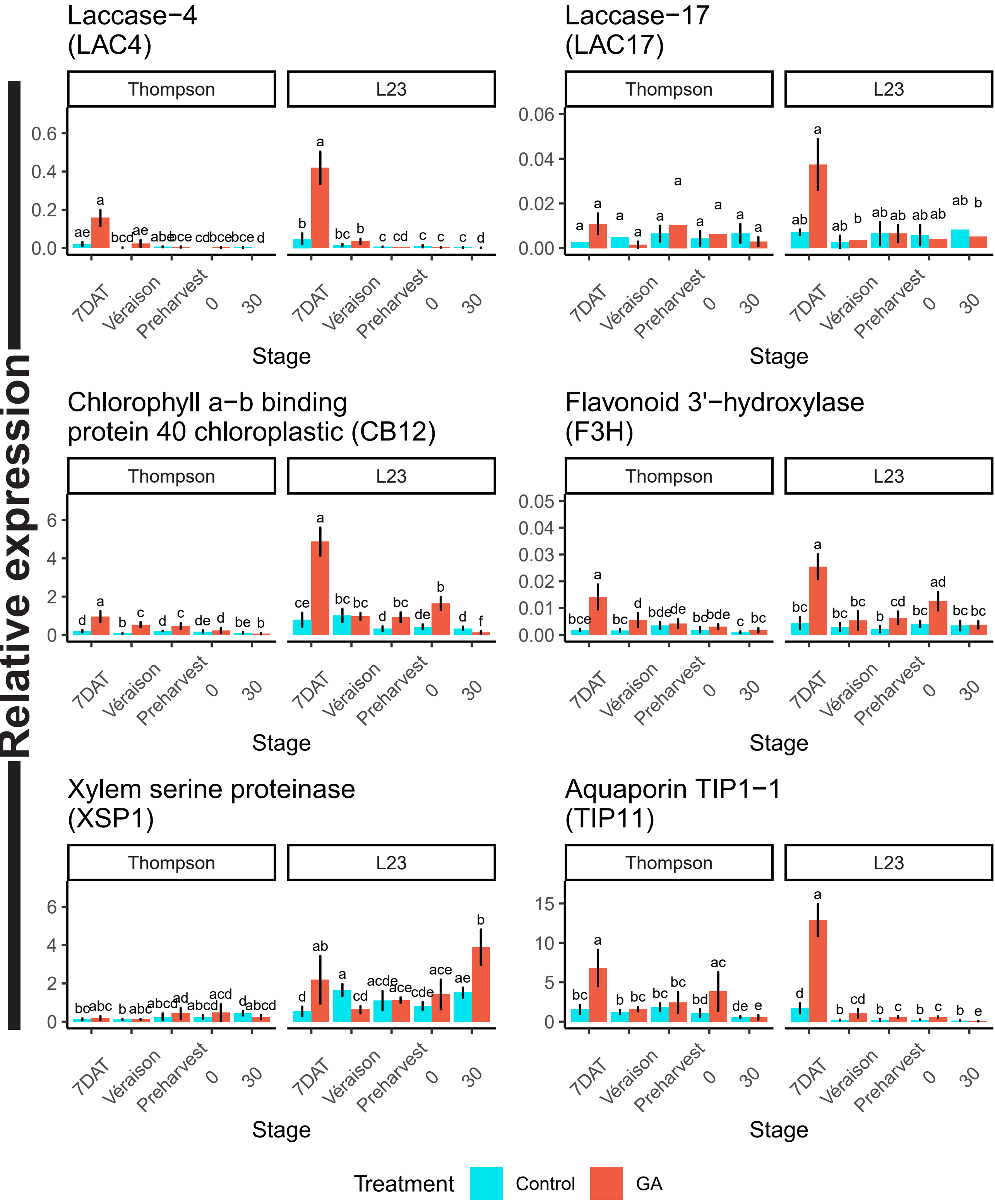

Relative expression
Laccase−4 (LAC4)
Laccase−17 (LAC17)
Chlorophyll a−b binding protein 40 chloroplastic (CB12)
Flavonoid 3'−hydroxylase (F3H)
Xylem serine proteinase (XSP1)
Aquaporin TIP1−1 (TIP11)
Thompson
L23
7DAT
Véraison
Preharvest
0
30
Stage
Treatment
Control
GA
